# Invader at the edge — Genomic origins and physiological differences of round gobies across a steep urban salinity gradient

**DOI:** 10.1111/eva.13437

**Published:** 2022-07-16

**Authors:** Leon Green, Ellika Faust, James Hinchcliffe, Jeroen Brijs, Andrew Holmes, Felix Englund Örn, Ola Svensson, Jonathan A. C. Roques, Erica H. Leder, Erik Sandblom, Charlotta Kvarnemo

**Affiliations:** ^1^ Department of Biological and Environmental Sciences University of Gothenburg Gothenburg Sweden; ^2^ Linnaeus Centre for Marine Evolutionary Biology University of Gothenburg Strömstad Sweden; ^3^ Gothenburg Global Biodiversity Centre University of Gothenburg Gothenburg Sweden; ^4^ Tjärnö Marine Laboratory, Department of Marine Sciences University of Gothenburg Strömstad Sweden; ^5^ Institute of Marine Biology University of Hawai'i Kaneohe Hawai'i USA; ^6^ Department of Educational Work University of Borås Borås Sweden; ^7^ Natural History Museum University of Oslo Oslo Norway

**Keywords:** biological invasions, euryhalinity, exotic species, osmoregulation, phenotypic sorting, seascape genomics

## Abstract

Species invasions are a global problem of increasing concern, especially in highly connected aquatic environments. Despite this, salinity conditions can pose physiological barriers to their spread, and understanding them is important for management. In Scandinavia's largest cargo port, the invasive round goby (*Neogobius melanostomus*) is established across a steep salinity gradient. We used 12,937 SNPs to identify the genetic origin and diversity of three sites along the salinity gradient and round goby from western, central and northern Baltic Sea, as well as north European rivers. Fish from two sites from the extreme ends of the gradient were also acclimated to freshwater and seawater, and tested for respiratory and osmoregulatory physiology. Fish from the high‐salinity environment in the outer port showed higher genetic diversity, and closer relatedness to the other regions, compared to fish from lower salinity upstream the river. Fish from the high‐salinity site also had higher maximum metabolic rate, fewer blood cells and lower blood Ca^2+^. Despite these genotypic and phenotypic differences, salinity acclimation affected fish from both sites in the same way: seawater increased the blood osmolality and Na^+^ levels, and freshwater increased the levels of the stress hormone cortisol. Our results show genotypic and phenotypic differences over short spatial scales across this steep salinity gradient. These patterns of the physiologically robust round goby are likely driven by multiple introductions into the high‐salinity site, and a process of sorting, likely based on behaviour or selection, along the gradient. This euryhaline fish risks spreading from this area, and seascape genomics and phenotypic characterization can inform management strategies even within an area as small as a coastal harbour inlet.

## 1 INTRODUCTION

Non‐native invasive species (NIS) rank as the fifth largest threat to biodiversity (WWF, 2020). The number of non‐native species translocated to novel habitats is increasing with growing global trade (Sardain et al., 2019). While the majority of these species do not become invasive (Jeschke & Strayer, 2006), the risk of occurrence is increasing with these introduction events (Sardain et al., 2019). The severity of a species invasion (i.e. the invader's population growth rate) is also expected to increase due to admixture effects through genetic variance from multiple source population (Barker et al., 2019; Keller et al., 2014; Keller & Taylor, 2010), as well as increased propagule pressure from transport vectors (e.g. via ballast tanks of ships). Although mitigation efforts are in place through the Ballast Water Management Convention of 2017, the pre‐COVID‐19 pandemic projection of shipping intensity still predicted an increase of up to 1000% in cargo vessel transports between 2018 and 2050 (Sardain et al., 2019). The consensus is that the risks of aquatic species invasions are increasing, with irreparable damage to many species, ecosystems and ecosystem services (Anton et al., 2019; Cuthbert et al., 2021). Knowing why certain NIS are successful in different environments is therefore key to understanding where the highest risk of spread can occur and where mitigation and control measures can be the most efficient.

Many NIS show high phenotypic plasticity (Davidson et al., 2011; Lande, 2015), and many of them can also exhibit strong local adaptation (Colautti & Lau, 2015; Moran & Alexander, 2014), even within the same introduced range. While aquatic environments, especially marine ones, differ from terrestrial environments with regard to their increased connectivity, marine seascape genomics (Selkoe et al., 2016) have shown strong genetic separation linked to environmental differences even at small spatial scales (Michalek et al., 2021; Westley et al., 2013). These differences are based on differences in the environment, such as biotic toxins (Wendling & Mathias Wegner, 2015), salinity (Caputi et al., 2019; Chen et al., 2021; Green, Apostolou, et al., 2021; Renborg et al., 2014) and/or temperature refugia (Tepolt & Palumbi, 2015). During aquatic species invasions, these environmental factors also present physiological barriers and gradients that can shape the process by which a species establishes (Christensen et al., 2021; Green, Niemax, et al., 2021), evolves (Green, Apostolou, et al., 2021), spreads (Magellan et al., 2019) and interacts with other species in their surrounding environment (Moyle & Light, 1996).

One such important environmental barrier is salinity. Organisms have adapted to the prevailing conditions in each of the two most common aquatic environments: freshwater and seawater. A consequence of this dichotomous evolution is that fishes that are able to move between salinities (e.g. a NIS expanding from an estuary) need to substantially change their physiology when doing so (Brijs et al., 2017; Sundh et al., 2014; Yang et al., 2009). While most fishes only tolerate relatively small changes in environmental salinity, euryhaline teleosts can tolerate and acclimate to a broad range of salinities. In freshwater, a dilute ionic and osmotic medium, fish must hyper‐osmoregulate to counter the continual loss of salts and entry of water across their permeable body surfaces. Mechanisms of hyper‐osmoregulation include active branchial and gastrointestinal ion uptake, and high glomerular filtration rates and urine flows, while minimizing drinking rate and renal salt loss (Evans et al., 2005; Perry et al., 2003). Conversely, in marine environments, fish hypo‐osmoregulate to counter the osmotic loss of water and diffusional gain of salts. Mechanisms of hypo‐osmoregulation include increased drinking rates, active gastrointestinal ion uptake to drive water uptake, and increased branchial and renal ion excretion (Genz et al., 2011; Grosell, 2011). The energetic costs associated with osmoregulation can be measured through an organism's standard metabolic rate (SMR): the minimum oxygen uptake required to maintain homeostasis and bodily functions in a resting and nondigesting state (Chabot et al., 2016; Clark et al., 2013). Moreover, an organism's maximum metabolic rate (MMR), which is the highest oxygen uptake possible for the organism (e.g. during intense activity), can also be limited by abiotic constraints (Clark et al., 2013; Norin et al., 2015). This limitation will be evident in the aerobic scope (AS), that is, the potential increase in metabolic capacity (Fry, 1947; Fry & Hart, 1948). It has been hypothesized that growth limitations in suboptimal conditions result from reductions in AS, which would indicate a reduced bioenergetic capacity and the inability to supply oxygen sufficiently to sustain increased oxygen demand in such conditions (Clark et al., 2013; Pörtner & Farrell, 2008; Sandblom et al., 2016). Environmental stress can induce varying changes in metabolic rates (i.e. SMR, MMR and AS; Halsey et al., 2018), and if abiotic factors such as salinity negatively affect the AS, the establishment of a NIS in a novel environment can be predicted based on this (Behrens et al., 2017).

Since locally adapted subpopulations of NIS can differ in their ability to function in a novel environment (Green, Apostolou, et al., 2021), their genomic ancestry is also of importance to predicting future colonization patterns of a recently established species. For example, a NIS population with very limited genetic diversity might become well adapted to a particular area through a strong selective bottleneck effect, but fail to spread into adjacent unsuitable habitats due to trait divergence (Rius et al., 2015). In contrast, in a port with shipping connectivity to many areas where a NIS has become established, multiple introductions of divergent lineages and genetic admixture (Blakeslee et al., 2010; la Rue et al., 2011) can lead to novel trait combinations, which can potentially facilitate colonization and further adaptations (Qiao et al., 2019; Rieseberg et al., 1999; Rieseberg et al., 2007; Stelkens & Seehausen, 2009). This is exemplified in an NIS hybrid sculpin, which does not only inhabit novel habitats but also exhibits novel gene expression, compared with its parental species *Cottus perifretum* and *Cottus rhenanus*. These changes in the transcriptome appear to have been modified and optimized after the initial genome‐wide admixture (Czypionka et al., 2012). Alternatively, multiple introductions of genetically unique subpopulations could result in phenotypic (Shine et al., 2011) and genotypic sorting (Richardson & Urban, 2013). This phenomenon, where uniquely adapted or exapted subpopulations spread into the habitat where their phenotype's fitness is high, is expected to occur in environments with strong environmental gradients (Chevin & Lande, 2013; Ghalambor et al., 2007).

A NIS that often appears in areas with strong environmental gradients is the euryhaline round goby (*Neogobius melanostomus*, Pallas) (Christoffersen et al., 2019; Green et al., 2020; Kornis et al., 2012). The round goby is a benthic fish that belongs to a clade of Ponto‐Caspian gobies (Agorreta et al., 2013; Thacker, 2015), many of which have adapted to both brackish and freshwater environments and, as a result, have the potential to survive and reproduce in very different environmental conditions (Kornis et al., 2012). Many species in this clade are also considered invasive (Stepien & Tumeo, 2006), and the round goby is well known to have severe effects on ecosystems through predation mainly on the local invertebrate fauna (Van Deurs et al., 2021). Salinity has been proposed to affect individual growth patterns for the species, due to osmoregulatory costs (Behrens et al., 2017; Kornis et al., 2012). Round gobies from brackish waters have been shown to be larger on average than fish in freshwater (Kornis et al., 2012). Age at sexual maturity has also been estimated to be one year less in the fresh water Great Lakes region compared with the native range (MacInnis & Corkum, 2000), though there are currently no explanations as to why (Kornis et al., 2012). Recently, sperm function in different salinities was strongly linked to the genetic divergence in the species within its European distribution, pointing towards local adaptation to salinity in both introduced and native populations (Green, Apostolou, et al., 2021).

**FIGURE 1:**
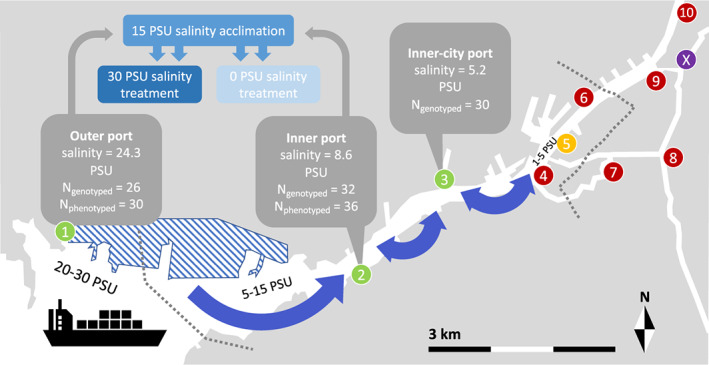
Infographic of study design and scenarios investigating the spread of the invasive round goby (*Neogobius melanostomus*) across the steep salinity gradient of the Port of Gothenburg, Sweden. The three sites yielding genotypic data (green circles 1, 2 and 3), and the two of those sites yielding phenotypic data (1 and 2) are marked with grey info boxes. Numbered circles mark sites where initial sampling occurred in 2016: green shows sites where fish were caught for the study, yellow where one fish was caught and excluded, and red where no fish were caught after a minimum of 6 fishing hours with baited hook and line. Sites 7 and 10 were also fished with 3 baited cages overnight. An eleventh site (off the map 1 km up the river) was also sampled without catch. The purple circle marked X shows the site furthest upstream where *N. melanostomus* has been found during a fishing survey in 2018 (not part of the present study). Habitats are roughly categorized and separated with dotted lines according to salinity. Striped polygon shows the extent of the Gothenburg international shipping port, Scandinavia's largest port with over 5300 cargo vessels visiting in 2020

One site of introduction, the Port of Gothenburg, situated on the Swedish west coast, is unique in a global perspective. At this site, the brackish round goby is recorded in the highest known salinity (29 PSU at the time of this study, now likely higher due to continuing expansion). This area is a marine coastal environment, with fluctuating salinity due to the Baltic Surface Current (Snoeijs‐Leijonmalm et al., 2016) running northwards along the coast, as well as input from several streams and rivers, in particular the large Göta Älv river. The Port of Gothenburg is situated at the rivermouth of Göta Älv (Figure 1). The river creates a steep salinity gradient in the urban Port of Gothenburg, where the round goby was first recorded in 2010. Since its first appearance, the species has increased in abundance and expanded from the urban port and out into the adjacent archipelago, with records of the species at 25 km northwards and 12 km southwards along the coast from the site of introduction (SLU Swedish Species Information Centre, 2022). Through shipping trade, the Port of Gothenburg is connected to several regions where the round goby is widespread, in particular, the western Baltic Sea, the northern Baltic Sea, and two large rivers in northern continental Europe, Elbe and Rhine (Azour et al., 2015; Puntila et al., 2018).

### 1.1 Aims

We had three aims: the first (1) was to understand the origin and connectivity of round gobies recently established in the Port of Gothenburg. We tested the hypothesis that the Port of Gothenburg would most likely originate from the spatially closest populations in the western Baltic Sea, by comparing single nucleotide polymorphisms (SNPs) in gobies collected from three sites in the Port of Gothenburg with fish sampled in the western Baltic Sea, the central Baltic Sea, and two rivers in northern continental Europe. The second (2) hypothesis we wanted to test was that fish in the low‐salinity inner port were genetically different and/or of different origin compared with fish in the high‐salinity outer port, which would indicate a process of sorting or selection in the different environments. Our third hypothesis (3) was that fish from the outer and inner part of the port would differ in their ability to acclimate to (and therefore spread in) seawater (30 PSU) and freshwater (0 PSU). We tested this by acclimating fish from two sites of different salinities to these conditions (as well as an optimal ‘baseline’ brackish 15 PSU condition) and then tested their osmoregulatory capacity and physiology.

## 2 METHODS

### 2.1 Sampling sites

Research on live fish was conducted under ethical permit nr 86–2013 issued by the Ethical Committee for Animal Research in Gothenburg. Eleven localities were sampled during September and October of 2016 while monitoring the species' distribution along the salinity gradient that exists within the Port of Gothenburg (Figure 1). Fish were caught using hook and line, as well as overnight Ahti traps, baited with frozen–thawed shrimp for both methods. Three locations to the west of the Gothenburg Port yielded sufficient sample sizes for population genetic analysis. The highest salinity site, the Outer port (57°41′41.1″N 11°48′57.5″E), had an average salinity of 24.3 ± 5.2 PSU (6 samples) with measurements reaching as high as 29.5 PSU. Specimens at this site were caught between the 6 and 20 October 2016. Sufficient fish were also caught for experimental purposes at a site with low salinity: an Inner port site (57°41′22.5″N 11°54′05.2″E) with an average salinity of 8.6 ± 0.4 PSU (6 samples) (i.e. 15.7 PSU lower than the above mentioned). Specimens at this site were caught between the 26 and 27 September 2016. A third location, the Inner‐city port (57°42′10.4″N 11°55′39.5″E), was sampled for genomic measurements on the 3 September 2016. This site had an average salinity of 5.2 ± 0.5 PSU (2 samples). All salinity measurements (Table S1) were taken from water close to the bottom of the fishing areas (roughly 3–4 m below the surface) and taken with a multimeter (HQ30d, Hach, Loveland, USA).

### 2.2 Extracting DNA

Caudal fins from fish used in the experiments described below, as well as fish not used in the experiments, were sampled from euthanized individuals (from the experimental animals, see below; for the Inner‐city port, by blunt trauma and destruction of the brain with a scalpel) and individually stored in 99.7% ethanol at −15°C (up to 936 days) before extractions took place. Before DNA was extracted, ethanol‐stored fin clips were air‐dried and weighed to ensure the dried tissue weight was between 100 and 250 μg. Extractions occurred according to the manufacturer's protocol with minor adjustments outlined in Green, Apostolou, et al. (2021) (Qiagen DNeasy Blood & Tissue Kit). DNA concentrations were checked using a fluorometer (Qubit Fluorometer 3.0, Thermo Fisher Scientific, Waltham, USA). Each sample was then diluted to 100 ng/μl using laboratory‐grade nuclease‐free water.

### 2.3 Creating genomic libraries

In total, five separate libraries were sequenced in 2017 and 2019, which included samples from other European round goby populations (Green, Apostolou, et al., 2021). Library preparation followed a genotyping‐by‐sequencing (GBS) protocol (Elshire et al., 2011). For library preparation, 100 ng of DNA of each individual was digested using 1.5 μl of restriction enzyme (Pst1 HF 20 U/ml, New England Biolabs) and 2 μl of Cut Smart Buffer in a 20 μl reaction in a thermocycler at the following program: 1 h at 37°C; 15 min at 75°C; and 10 min at 4°C. Each individual was ligated to a unique forward adapter (barcode) and a common reverse adapter (1 μl of 50 nM pooled forward and reverse adapters), along with 21 μl dH_2_O, 5 μl T4‐buffer and 3 μl T4‐ligase (New England Biolabs). The ligation ran for 1 h at 22°C, then stopped for incubation at 30 min at 65°C and was then cooled down to 4°C. Fifteen microlitres from each barcoded sample was then pooled into libraries of 95 individual samples together with a blank control. To avoid potential biases during sequencing, individuals from the same population were distributed across multiple libraries. Cleaning and concentration of the libraries were done using AMPure XP beads (Beckman Coulter), and then, the pool was amplified with the following components: cleaned ligation pool (23.5 μl), 1.5 μl primer mix (12.5 μM) and 25 μl KAPA HIFI Hot start 2X Mastermix at the following program: 5 min at 72°C; four cycles of (30 s at 95°C; 10 s at 95°C; 30 s at 65°C; and 30 s at 70°C); 13 cycles of (10 s at 95°C; 30 s at 65°C; and 20 s at 72°C); 5 min at 72°C; and 10 min 4°C. After amplification, each library was again cleaned with AMPure XP beads. Size selection of 290–390 bases was run in duplicate using a BluePippin (Sage Biosciences, Beverly, USA) with 2% gel cassette and V1 marker. A final cleaning and pooling of the two size‐selected elution per library was done with AMPure XP beads and eluted in 10 mM Tris–HCl. The final library size range was confirmed on a Fragment Analyzer (Agilent) with a high‐sensitivity genomic DNA kit (DNF‐488, Agilent) to a mean size range between 350 and 380 bases.

### 2.4 Generating genomic markers and filtering

Sequencing of the five libraries was performed at the Beijing Genome Institute (China) using the Illumina HiSeq 4000 platform (100 PE). Libraries were demultiplexed and quality‐trimmed using Cutadapt v2.10 (Martin, 2011). The sequences were analysed in conjunction with other European populations (Green, Apostolou, et al., 2021). To allow for sufficient overlap during mapping, only read pairs where both reads contained more than 50 bases were kept in the analysis. Paired‐end reads were aligned to the round goby reference genome (Ensembl release 100, REF) using Bowtie2 v2.3.5.1 (Langmead & Salzberg, 2012) for each individual. Conversion of the sequence alignment map files to sorted binary version files was done using SAMtools v1.10 (Li et al., 2009), and variant calling and filtering was done using bcfools mpileup, call and filter functions (v1.10.2) (Li, 2011). Sites at which bcftools identified multiple variant types (single nucleotide polymorphisms (SNPs), indels and multi‐base polymorphisms) were removed. If an individual had more than 0.25 loci with no reads, it was also removed from the data set. SNPs were filtered for read depth of 6 and a minimum genotype quality of 30 and set to 0 (./.) if this was not met. Bcftools and Plink v1.07 (Purcell et al., 2007) were used for the final filtering to remove any SNPs with hwe of less than 0.001 (separately at each site), missing genotypes ≥0.1, minor allele frequency <0.01 or heterozygosity >0.75. Of these nine external sites analysed together with the three sites from the Port of Gothenburg, two were from European rivers (0 PSU), four were from the western Baltic Sea (10–15 PSU), and three were from the central and northern Baltic Sea (2–5 PSU) (Figure S1). The final data set contained a total of 305 individuals sampled from 12 different geographic sites, and genotyped at 12,937 polymorphic SNPs with 2.5% missing data.

### 2.5 Genomic analysis

Genomic analysis was carried out in R 4.1.2 (R Core Team, 2021). Genetic diversity and divergence estimates were calculated with the R package diveRsity (Keenan et al., 2013). *F*_ST_ was estimated for each population pair (Weir & Cockerham, 1984). Fisher's exact probability test was used to assess statistical significance of *F*_ST_ estimates, followed by false discovery rate correction (Benjamini & Hochberg, 1995) to correct for multiple testing. Divergence and distribution of variation was assessed with an analysis of molecular variance (AMOVA) among the four regions (Port of Gothenburg, western Baltic Sea, central and northern Baltic Sea and European rivers), sampling sites and among sampling sites within regions using the R package poppr (Kamvar et al., 2014).

Individual differentiation and ancestry was estimated and visualized using two different clustering methods, first using the ‘snmf’ function in the R Package LEA (Frichot & François, 2015), and second with a principal component analysis (PCA) in the R package ade4 (Chessel et al., 2004; Dray et al., 2007). LEA estimates individual ancestry by utilizing a sparse non‐negative matrix factorization algorithm (sNMF) to compute least‐squares estimates of ancestry coefficients. The ancestry coefficients are estimates of how much of each individual genome originated from a specified number of ancestral populations *K*. The number of *K* ancestral populations that best explains the data ancestry was selected based on the lowest cross‐entropy criterion (CEC). The coefficients were estimated by running 6 replicates of *K* 1–20. PCA is a multivariate exploratory approach that makes no prior assumptions about the number of groups or clusters. Allele frequencies were centred but not scaled, and missing data were replaced by mean allele frequencies with the function scaleGen in the R package adegenet (Jombart, 2008; Jombart & Ahmed, 2011).

Two approaches were used to detect outlier loci putatively under selection: the FST‐based approach OutFLANK (Lotterhos & Whitlock, 2014), which infers the distribution of neutral loci from a trimmed FST distribution to then calculate each SNP likelihood to be an outlier, and the PCA approach pcadapt (Privé et al., 2020), which is an unconstrained ordination method that uses principal components to define the underlying clustering prior to the outlier scan. In both OUTFLANK and pcadapt, outlier SNPs were identified using the default settings. The number of K principal components in pcadapt was based on the best CEC identified in the sNMF analysis. For both approaches, a q‐value (FDR corrected p‐values) of 0.05 was used as a threshold for statistical significance. SNPs that were identified as significant outliers by both approaches were considered putatively under selection and were removed from the data to create a data set of putatively neutral SNPs. Following this, the above genomic analyses were re‐run on only neutral SNPs to avoid confounding neutral measures of population divergence with patterns generated by selection.

### 2.6 Animal husbandry

Since the two westernmost sites (Outer port and Inner port) had the highest catch per unit effort, animals from these sites were kept for experimental purposes. After catch (at the above mentioned dates) live fish were transported in a cooling box filled with water from the sampling site and aerated using a battery driven pump for <1 h until placed in aquaria set up in the aquarium facility in the Zoology building at the Department of Biological and Environmental Sciences, University of Gothenburg, Medicinaregatan 18A, Gothenburg, Sweden. Animals were kept in one of four systems of three connected and recirculating aquaria of 210 L (120 × 35 × 55 cm), each equipped with a foam filter with air‐driven circulation along with an air stone. The third tank, placed beneath the other two, acted as a sump, hosting a filter that water from the above tanks flowed through before reaching the pump, circulating it to the top tank where it reached a Vecton V2 600 Ultraviolet Water Steriliser (Tropical Marine Centre, Hertfordshire, UK) fitted with a 25‐W G25T8 UV Lamp to further clean the water. Water in the bottom tank was replaced every week, and filters were cleaned in order to keep nutrient levels low. Ammonia, nitrite and nitrate levels were tested for weekly and never reached above 0, 0 and 20 ppm respectively. The tanks were fitted with 2 cm of 1‐ to 5‐mm gravel as substratum and 4 halved clay pots and 3 rocks to create a habitat featuring multiple hiding spots for the fish. In each closed system, two of the three tanks hosted 7–10 fish, while the bottom was left empty (Figure S2).

At first introduction to the tank, fish were drip‐acclimated (over 1 h) to a salinity of 15 PSU. Water temperature was determined by the air temperature of the room, which was set to 11 °C. For ease of husbandry, the light cycle was set to a 12‐h light: 12‐h dark photoperiod using 4 separate 58 W t5 fluorescent lights installed in the ceiling in the middle of the room and dimmed to 80% intensity. Fish were fed ad libitum once daily with 5 g of 2‐mm fish pellets (size 2 spirit trout 600‐40A 7 SE, Skretting, Stavanger, Norway) and 3 g of frozen–thawed krill (*Euphasia pacifica*) (Krill Pacifica, Ocean Nutrition, Essen, Belgium) per tank, except during weekends when they were fed 3 g of pellets per tank (roughly 1 g of food per fish and day). Leftover food was removed every weekday. After 112 ± 12 days, a total of 24 fish (12 from each site of catch) were tested for their metabolic performance according to the below described protocol (outlined under 2.8).

### 2.7 Acclimation to salinity treatments

In order to compare the potential physiological constraints of gobies moving up the river or out to sea, salinity was changed to 0 PSU for one half, and to 30 PSU for the other half of the fish caught from the Outer port and Inner port after the first measurements of metabolic performance at 15 PSU (see below). The salinity in the aquaria was gradually changed (lowered or raised) at a rate of 1 PSU per day over two weeks using artificial seawater from salt (Aquaforest Sea Salt, Aquaforest, Brzesko, Poland) and drinking grade tap water (see Supplementary Information for volumes), creating four different treatment groups: Outer port at 30 PSU (*N* = 15); Outer port at 0 PSU (*N* = 15); Inner port at 30 PSU (*N* = 18); and Inner port at 0 PSU (*N* = 18). After 30 ± 13 days in these conditions, six fish from each treatment group of similar mass were again tested for their metabolic performance. Average + max CI mass of fish from of the Outer port at 30 PSU was 43.8 ± 41.0 g; Outer port at 0 PSU was 43.2 ± 42.8 g; Inner port at 30 PSU was 40.8 ± 29.3 g; and Inner port at 0 PSU was 48.5 ± 25.3 gram. Sexes (males + females) of the tested fish were for the Outer port at 30 PSU = 4 + 2; Outer port at 0 PSU = 3 + 3; Inner port at 30 PSU = 2 + 4; and Inner port at 0 PSU = 2 + 4.

### 2.8 Measurement of oxygen consumption rate

Metabolism was measured using a cylindrical, intermittent flow‐through respirometer (volume of 0.584 L), which was submerged in a reservoir bath, containing flow‐through, aerated water similar to the treatment conditions in which the fish were held (Clark et al., 2013; Norin & Clark, 2016). Water was continuously circulated through each respirometer using an in‐line submersible pump within a recirculation loop, and the partial pressure of oxygen within the respirometer was measured continuously at 0.5 Hz using a FireSting O2 system (Pyro Science) calibrated in accordance with the manufacturer’s manual. Each respirometer was also equipped with an automated flush pump, which refreshed the water in the respirometers for a 5‐min wait period, after which a 10‐min recording period began of oxygen uptake began, ensuring that oxygen levels in the respirometer always remained above 90% air saturation.

Before being placed in the cylinders, fish from a randomly chosen treatment group were fasted for >24 h to avoid increased metabolism from digestion of food. Upon start, fish were individually taken out of the holding tank, measured and weighed to the nearest 0.1 gram. A manual chase protocol was selected as the method to elicit MMR, as this species can withstand high water velocities by attaching themselves to tank sides and bottoms using their fused pelvic fin ‘suction cup’, which prevents the use of a swim tunnel (Behrens et al., 2017). To measure MMR, each fish was placed in a circular tank (diameter 28 cm, water depth 15 cm) with ambient water from the treatment conditions in which the fish were held. In this circular tank, the fish were then manually chased for 5 min. All individuals were visibly exhausted by the end of the 5‐min period as highlighted by a lack of response to an experimenter tapping the caudal fin. Following the manual chase protocol, the fish was immediately placed into the respirometer, which was then sealed, and O2 was measured to determine MMR.

MMR was calculated from the first 2‐min decline in the partial pressure of oxygen within the respirometer, and RMR was calculated from the entire 10‐min decline using the following formula: whole animal oxygen uptake = [(*V*_r_ – *V*_f_) × ∆CwO_2_]/(∆*t* × *M*_f_), where *V*_r_ is the volume of the respirometer, *V*_f_ is the volume of the fish (assuming that the overall density of the fish is 1 g per ml of tissue; thus, *V*_f_ = mass of the fish, *M*_f_), ∆CwO_2_ is the change in the oxygen concentration of the water within the respirometer (CwO_2_ is the product of the partial pressure and capacitance of oxygen in the water, the latter being dependent on salinity and temperature), and ∆*t* is the time during which ∆CwO_2_ is measured (Clark et al., 2013). Since 21 out of 48 respirometry records showed a coefficient of variation of the mean of the lowest normal distribution of <5.6, the lowest 20% of O2 uptake measurements (q20) were used to calculate SMR (Chabot et al., 2016). Aerobic scope (AS) was calculated as MMR ‐ SMR, and Factorial AS (FAS) was calculated as MMR/SMR (Halsey et al., 2018).

### 2.9 Measuring blood and gill physiology

Five days after the final respirometry measurements were conducted, fish were euthanized and sampled for blood parameters and gill branchial Na^+^/K^+^ ‐ATPase (NKA) activity. The fish were fasted for 24 h prior to sampling to reduce variation on the blood and enzyme data related to digestion. On the day of sampling, all the fish from one tank were rapidly netted and kept together in a dark 10‐L bucket containing water from their tank. Fish were subsequently individually euthanized with a lethal dose of metomidate hydrochloride (Sigma‐Aldrich) (0.1 g/L). This procedure took less than 2 min.

Approximately 1 ml blood was withdrawn from the caudal vessels with a heparinized syringe fitted with a 25 Gauge needle. Two subsamples of blood in 80 μl heparinized microcapillary tubes centrifuged at 10,000 RCF (Relative Centrifugal Force) for 5 min in a Hct centrifuge (Haematokrit 210) were measured to determine haematocrit as the fractional red cell volume after centrifugation. The remainder of blood was immediately centrifuged at 1000 RCF for 5 min, and the plasma was stored at −80°C until further analyses.

Gill filaments were excised by lifting the operculum and removing 5–8 gill filaments from one gill arch. The filaments were stored in SEI buffer (150 mM sucrose, 10 mM EDTA, 50 mM imidazole; pH 7.4 with 0.1% Na deoxycholic acid) (Sigma‐Aldrich) and kept at −80°C for later determination of NKA enzymatic activity.

#### 2.9.1 Blood plasma analyses

Plasma concentration of cortisol was determined by radioimmunoassay as described in (Sundh et al., 2011) (modified from Young, 1986). Plasma osmolality was determined using a micro‐osmometer (Model 3320; Advanced Instruments). Total plasma potassium (K^+^), sodium (Na^+^) and calcium (Ca^2+^) were measured from whole plasma using a flame emission photometer, with LiCl as internal standard (Eppendorf AG, Hamburg, Germany; model ELEX 6361) (Sillanpää et al., 2016). Haemolymph glucose was measured with commercially available enzymatic test kit (Glucose HK; Sigma‐Aldrich), with protocols adapted to a 96‐well microplate (Schram et al., 2010). Samples were first diluted in distilled water (1:6), and 10 μl of the diluted sample or standard (5.55 mmol/L glucose) was mixed with 200 μl reagent provided with the kit and incubated for 15 min at 20°C. Absorbance was read within 30 min at 340 nm on a SpectraMax 190 Microplate Reader (Molecular Devices).

#### 2.9.2 Gill NKA activity

NKA activity was analysed using an NADH‐linked kinetic assay in a 96‐well microplate run at 25°C for 10 min, as described in McCormick (1996). Protein concentration of homogenates was determined using the Pierce BCA Protein Assay (Thermo Scientific). Both assays were run on a THERMOmax microplate reader using SOFTmax software (Molecular Devices).

### 2.10 Statistical analysis of the experimental results

Statistical tests of the fish's physiology were performed in R (version 3.6.2) using the packages car (Fox et al., 2013) and lme4 (Bates et al., 2015). For the metabolic rate measurements, the main predictor variables modelled as fixed effects were ‘site of catch’ (Outer port or Inner port) and ‘salinity treatment’ (0 PSU, 15 PSU, 30 PSU). To control for body mass, this variable was included in the model as a numerical predictor variable. These were tested in a full factorial design and simplified to fixed effects only when no interactions were found (Hendrix et al., 1982). The models were explored by visually inspecting the residuals vs fitted values, the frequency distribution of residuals, the theoretical and observed quantiles and high influence points, using the ‘plot(model)’ function. Variance inflation factors were analysed using the ‘vif(model)’ function, and none of concern were found. The data were normally distributed and met assumptions of sphericity. Sex was also included as random term in the model but was left out after no effect was found. Similarly, the factors ‘site of catch’ within the studied harbour (Outer port or Inner port) and ‘salinity treatment’ (0 PSU, 30 PSU) were tested for their effect on blood values and gill NKA activity. Again, assumptions and model variance inflation factors were tested as for the metabolic data described above, and no concerns relating to the applicability of the statistical tests were found.

## 3 RESULTS

### 3.1 Genetic diversity and divergence

Genetic diversity estimates showed only small differences between sites (Table S1). When compared to the other regions, the Port of Gothenburg demonstrated overall lower diversity in terms of allelic richness (Ar), per cent of polymorphic sites (P[%]), private alleles (Pa) and observed and expected heterozygosity (H_obs_, H_exp_). The only site with lower diversity was Mariehamn in the Central Baltic Sea. The highest genetic diversity among the Gothenburg sites was observed in the Outer port, which had 63.7% polymorphic sites, while the Inner port and the Inner‐city port only had 56.9% and 55.8%, respectively. The majority of private alleles were confined to the samples from the rivers Elbe (491) and Rhine (856). Most private alleles among the brackish sites were found in Turku (44), and none was found in the Port of Gothenburg when analysing each site separately. However, when comparing between regions, 19 private alleles were found in Gothenburg, 78 in the Central‐Northern Baltic Sea, 80 in the western Baltic Sea and 1868 in the European rivers.

**FIGURE 2:**
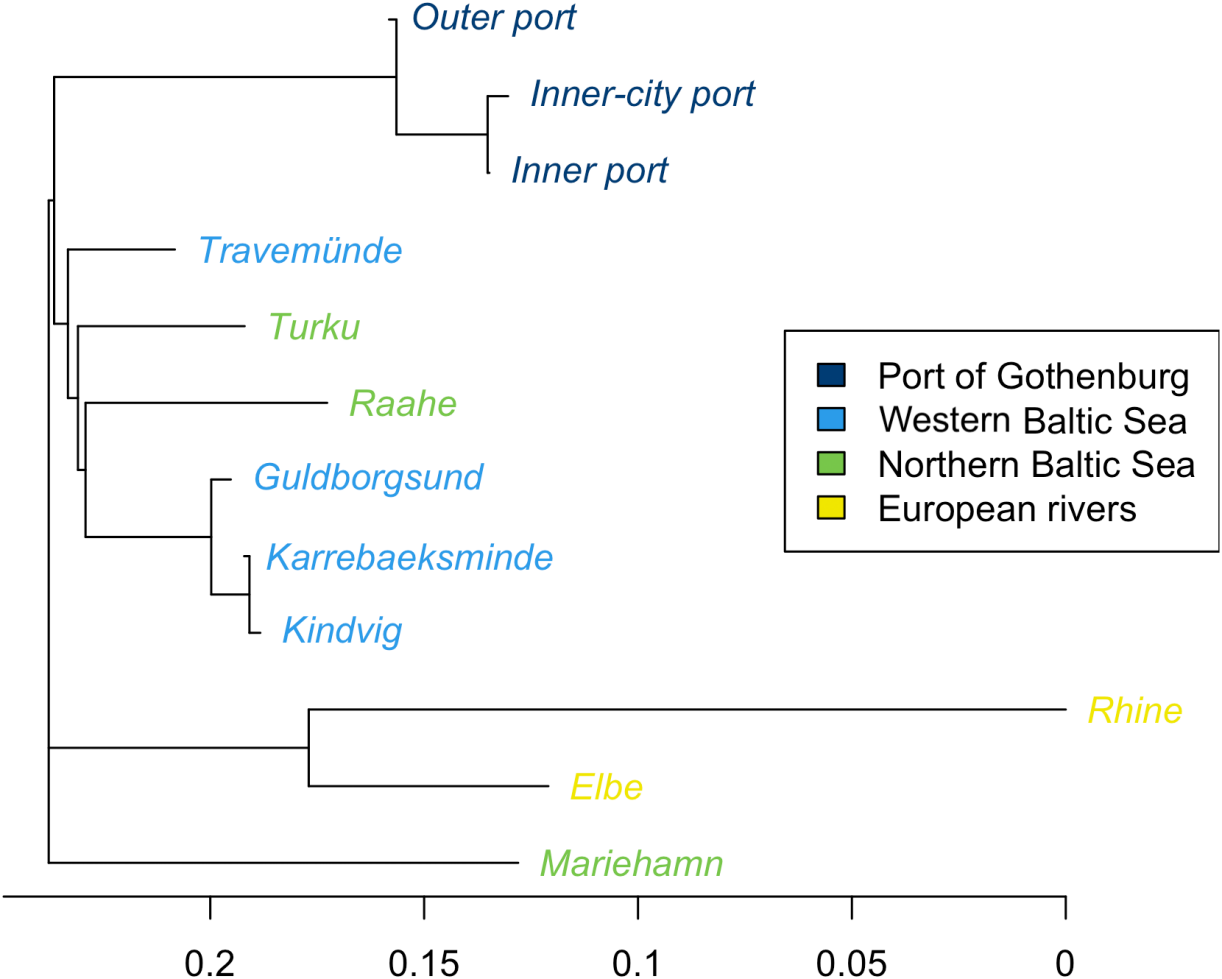
Neighbour‐joining tree based on pairwise *F*_ST_ estimates of round gobies (*Neogobius melanostomus*) sampled from 12 sites and genotyped at 12,937 SNPs. Colours represent the four larger geographic regions targeted

Estimates of pairwise *F*_ST_ showed that most sites were significantly differentiated from each other (Figure 2, Table S2). The smallest divergence among sites within each region was observed in the Port of Gothenburg (*F*_ST_ ~ 0.017), followed by western Baltic Sea (*F*_ST_ ~ 0.038), the central and northern Baltic Sea (*F*_ST_ ~ 0.14) and finally the European rivers (*F*_ST_ ~ 0.23). All the sites in the Port of Gothenburg were differentiated from all other sites in all the regions (*F*_ST_ ~ 0.109–0.336). Among the Gothenburg sites, only the pairwise comparison between the Outer port and the Inner‐city port was significant (*F*_ST_ = 0.0236). Within the Port of Gothenburg, there was almost 4 times higher divergence between the two inner ports and the ‘outer port’ (*F*_ST_ ~ 0.022) than between the inner port and the inner‐city port (*F*_ST_ = 0.005). Gothenburg Outer port was overall less divergent (*F*_ST_ ~ 0.13) from the Baltic Sea sites compared to the Inner port (*F*_ST_ ~ 0.16) and the Inner‐city port (*F*_ST_ ~ 0.16). However, despite the overall higher divergence in the Inner port, all three sites were more similar to the western Baltic Sea than to any other regions. Furthermore, all three sites showed the lowest divergence from Travemünde (*F*_ST_ = 0.109–01295). Analysis of molecular variance showed significant differentiation at all the tested levels (Table S3). Largest variation was explained by within individual variation (79.2%), followed by regions (9.8%), sites within regions (8.9%) and finally among individuals within sites (2.1%).

### 3.2 Clustering and assignment

**FIGURE 3:**
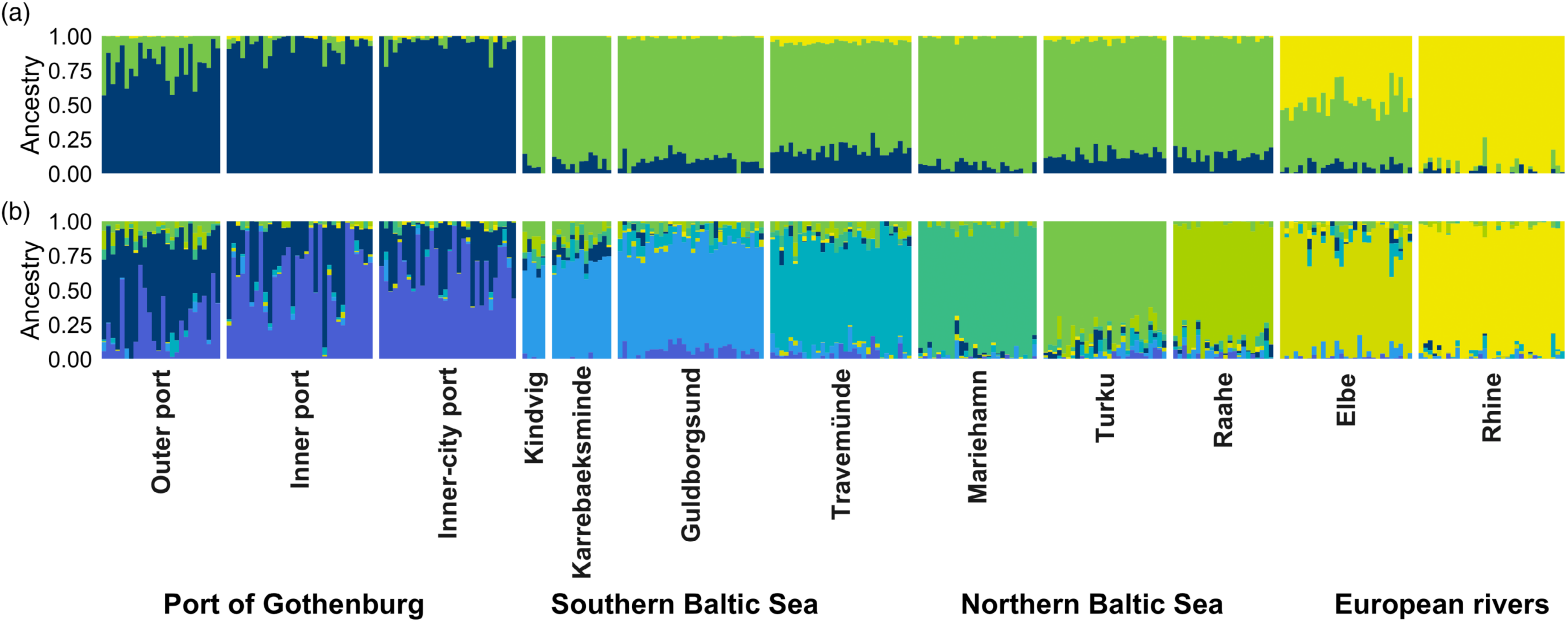
Individual ancestry of 305 round gobies (*Neogobius melanostomus*) based on 12,937 SNPs for (a) *K* = 3 and (b) *K* = 9 estimated using sNMF. Each vertical bar is one individual, and the colour is the proportion of that individual assigned to the different *K* clusters. Individuals are separated by sampling sites and grouped in the four Baltic Sea regions. Clusters 2–10 can be found in Figure S4

Individual assignment was estimated for a range of K clusters with sNMF, and was evaluated by visual inspection and their relative cross‐entropy criterion (Figure S3). The European rivers separate from the other sites already at *K* = 2. However, all the individual samples in the Elbe assigned 50: 50 to the two different clusters, suggesting a closer relationship of fish from Elbe to the Baltic Sea and Gothenburg populations, compared to fish from Rhine. At *K* = 3, all individuals in the three Gothenburg sites were assigned to a separate cluster (Figure S4). In concordance with *F*_ST_ estimates, individuals sampled in the Outer port were more similar to the brackish cluster than the two ‘inner port’ sites, and assigned to a small percentage (~25%) to the same cluster as the Baltic Sea. With increasing *K* clusters, site after site separated from the larger Baltic Sea cluster. There were little signs of admixture and no individuals with clear assignment to any clusters other than their own site. The only sites that did not show any distinct separation were the three sites in the western Baltic Sea, Kindvig, Karrebaeksminde and Guldborgsund. Some individuals in Gothenburg, mainly from the Outer port, started to separate from other Gothenburg samples at *K* = 9 (Figure 3b), which was the K with lowest cross‐entropy, and thus best supported.

**FIGURE 4:**
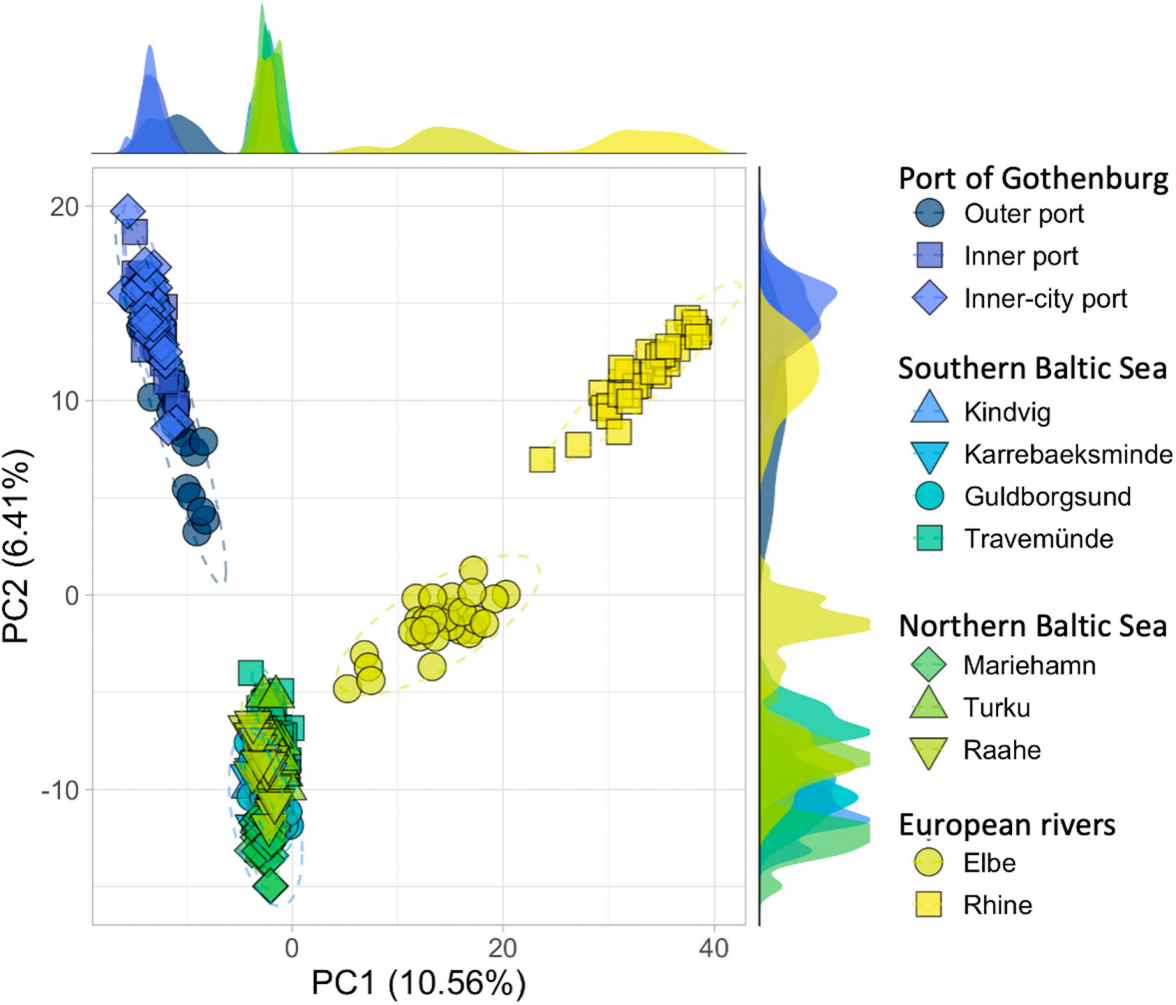
First (*x*‐axis) and second (*y*‐axis) component of a principal component analysis (PCA) on 305 round gobies (*Neogobius melanostomus*) genotyped at 12937 SNPs. The first component explains 10.56% of the total variation and the second 6.41%. Each point represents one individual, colours represent sampling sites, and shape is used for better distinction

The principal component analysis (PCA) revealed similar clustering as seen in the individuals' ancestry clustering. The first two principal components (PC) explained ~17% of the total variation (Figure 4). While the first PC clearly separated Gothenburg, Baltic Sea, Elbe and Rhine from left to right, PC2 clustered Gothenburg and Rhine away from the Baltic Sea with Elbe in the middle. On the third and fourth PC, which together explained over 6.5% of the variation, Mariehamn and the Elbe separated out from the other Baltic Sea samples, first in the same and then in opposite directions (Figure S5). Succeeding components (PC5–PC7) showed separation among Baltic Sea samples, and together explained just under 5% of the total variation. Consecutive PCs all had less than 1% explanatory power and thus were not investigated further. Individuals sampled in Gothenburg's Outer port showed a larger spread and separation than individuals in the Inner port on both the first two components. This was also visible when performing a PCA only on individuals sampled in Gothenburg (Figure S6).

The two outlier approaches, pcadapt and OutFLANK, did not identify any overlapping outlier loci. When analysing all samples, pcadapt found 395 putative loci under selection, while OutFLANK did not detect any. The PCA and sNMF analysis described above were repeated without the 395 outliers identified by pcadapt, which ensured that these loci did not alter the result (not shown). However, as no SNPs were identified as significant outliers by both methods, no SNPs were removed from the final data set.

### 3.3 Metabolic performance

Standard metabolic rates (SMRs) between the two sites (mean Inner port SMR in 15 PSU: 34.04 mg O_2_ h^−1^ kg^−1^, CI: 30.8–37.3; mean Outer port SMR in 15 PSU: 32.5 mg O_2_ h^−1^ kg^−1^, CI: 28.8–36.3) were not affected by site of catch (lm, site, *F*_3,44_ = 0.656, *p* = 0.423, Adj. *R* ^2^ = 0.030) and were not affected by the salinity treatment (lm, salinity treatment, *F*_3,44_ = 1.890, *p* = 0.163, Adj. *R* ^2^ = 0. 030). No interaction effects on SMR were found.

Maximum metabolic rate (MMR) differed between fish from the two sites, Inner and Outer port, in Gothenburg (lm, site, *F*_3,44_ = 3.92, *p* = 0.00285, Adj. *R* ^2^ = 0.157), with higher MMR for fish caught from the high‐salinity Outer port site (mean MMR in 15 = PSU: 166.9 mg O_2_ h^−1^ kg^−1^, CI: 143.4–190.4) compared with the Inner port site (mean MMR in 15 PSU: 143.4 mg O_2_ h^−1^ kg^−1^, CI: 130.4–156.4). MMR was not affected by either the 0 PSU treatment (10.2 mg O_2_ h^−1^ kg^−1^, CI: −8.2 ‐ 28.6) or the 30 PSU treatment (−2.75 mg O_2_ h^−1^ kg^−1^, CI: −21.1–15.6) (lm, salinity treatment, *F*_3,44_ = 0.887, *p* = 0.412, Adj. *R* ^2^ = 0.157). Fish of lower body mass from the Outer port site were found to have higher MMR (lm, site * body mass, *F*_3,44_ = 6.107, *p* = 0.013, Adj. *R* ^2^ = 0.246).

The higher MMR of the Outer port fish carried over onto the aerobic scope, with a higher scope for fish from this site (AS 15 PSU 135.1 mg O_2_ h^−1^ kg^−1^, CI: 104.7–163.9) compared to the Inner port (AS 15 PSU 109.4 mg O_2_ h^−1^ kg^−1^, CI: 96.5–122.2), and site therefore had significant effect on this measurement (lm, site, *F*_3,44_ = 11.619, *p* = 0.001, Adj. *R* ^2^ = 0.169). There was no effect of the salinity treatment (lm, salinity treatment, *F*_3,44_ = 0.472, *p* = 0.627, Adj. *R* ^2^ = 0.169), and similar to the MMR values, fish of lower body mass were found to have higher AS (lm, site × body mass, *F*_3,44_ = 6.597, *p* = 0.018, Adj. *R* ^2^ = 0.263).

**FIGURE 5:**
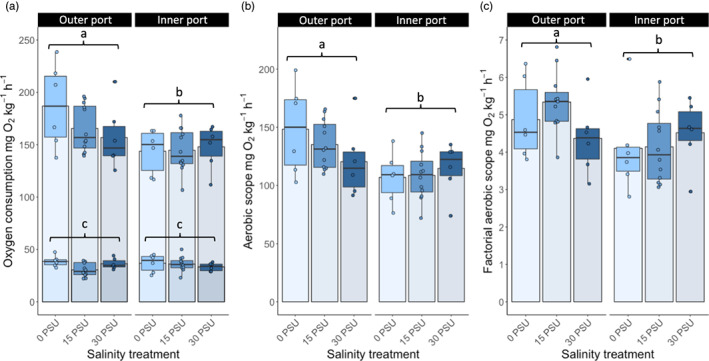
Metabolic performance of round goby (*Neogobius melanostomus*) caught from two sites of different ambient salinities (Outer port or Inner port) and acclimated to salinities of first 15, then either 0 or 30 PSU at 10°C. Bars show mean values, boxes show median, and upper and lower quartile, and error bars show max and min with outliers denoted by dots. Jittered points show individual values. Letters indicate statistical differences outlined in the results. Panels show the following: (a) oxygen uptake (mg O_2_ kg^−1^ h^−1^) shown in top values as maximum metabolic rate (MMR) and shown in bottom values as resting metabolic rate (SMR) (lowest 20% of values measured every 15 min over 48 h). (b) Aerobic scope (MMR – SMR). (c) Factorial aerobic scope (MMR/SMR)

The higher MMR of the Outer port fish was also seen in the factorial AS, where site had a significant effect (lm, site, *F*_3,44_ = 7.754, *p* = 0.008, Adj. *R* ^2^ = 0.120). There was no effect from the salinity treatment (lm, salinity treatment, *F*_3,44_ = 0.816, *p* = 0.449, Adj. *R* ^2^ = 0.120), and no interaction effects on factorial AS were found (Figure 5).

### 3.4 Blood parameters and gill NKA activity

Blood samples from the fish kept in 0 and 30 PSU (Outer port 0 PSU *N* = 11; Outer port 30 PSU *N* = 9; Inner port 0 PSU *N* = 12; Inner port 30 PSU *N* = 10) showed some differences in certain traits (see Table 1 for a summary). Fish from the Inner port had higher haematocrit levels (lm, site, *F*_2,38_ = 9.380, *p* = 0.004, Adj. *R* ^2^ = 0.187) and plasma calcium content (lm, site, *F*_2,35_ = 10.037, *p* = 0.003, Adj. *R* ^2^ = 0.179) than fish from the Outer port site. The site of catch did not affect any other blood parameter. Blood osmolality (lm, salinity treatment, *F*_2,38_ = 6.455, *p* = 0.015, Adj. *R* ^2^ = 0.122) and Na^+^ content (lm, salinity treatment, *F*_2,35_ = 5.368, *p* = 0.027, Adj. *R* ^2^ = 0.090) were higher in fish kept in 30 PSU than fish kept in 0 PSU. Cortisol was higher in fish from the 0 PSU treatment (lm, salinity treatment, *F*_2,31_ = 7.856, *p* = 0.009, Adj. *R* ^2^ = 0.192) compared to the 30 PSU treatment. The salinity treatment did not affect any other blood parameter. No interaction effects between site of catch and salinity treatment were found for any blood parameters.

**TABLE 1:** Statistical results of the effects on tissue parameters associated with osmoregulatory capacity in teleost fish, sampled from round goby (*Neogobius melanostomus*)

Tissue	Parameter	Predictor	*B*	SE *B*	CI	*p*
Gill	Gill NKA	Site	−0.076	1.005	−2.12 to 1.97	0.940
		Salinity treatment	0.513	1.002	−1.52 to 2.55	0.612
Blood	Osmolality	Site	3.647	3.524	−3.45 to 10.70	0.305
		Salinity treatment	−8.824	3.524	−16.00 to −1.69	0.017*
Blood	Potassium (K^+^)	Site	0.0610	0.152	−0.25 to 0.37	0.690
		Salinity treatment	−0.072	0.153	−0.38 to 0.24	0.641
Blood	Sodium (Na^+^)	Site	1.881	3.505	−5.23 to 9.00	0.595
		Salinity treatment	−7.893	3.520	−15.0 to −0.75	0.031*
Blood	Calcium (Ca^2+^)	Site	0.406	0.128	0.15 to 0.67	0.0032***
		Salinity treatment	0.042	0.129	−0.22 to 0.30	0.74681
Blood	Haematocrit	Site	4.469	1.459	1.51 to 7.42	0.004***
		Salinity treatment	2.137	1.466	−0.83 to 5.10	0.15324
Blood	Cortisol	Site	12.206	8.684	−5.50 to 29.9	0.16977
		Salinity treatment	24.787	8.562	7.32 to 42.2	0.007**
Blood	Glucose	Site	−0.215	0.355	−0.94 to 0.51	0.549
		Salinity treatment	0.391	0.361	−0.34 to 1.12	0.287

*Note*: Fish were caught from two sites of different ambient salinities (Outer port—24.3 PSU, or Inner port—8.6 PSU) and acclimated to salinities of either 0 or 30 PSU at 10°C for 47 days. Intercept of models aligns at Outer port (Site) and 30 PSU (Salinity treatment). Significance bold values are highlighted in bold: *p < 0.05, **p < 0.01 and ***p < 0.005. Complete data of group means are available in Table S4.

Gill NKA was not affected by site of catch (lm, site, *F*_2,33_ = 0.147, *p* = 0.704, Adj. *R* ^2^ = −0.056) or the salinity treatment (lm, salinity treatment, *F*_2,33_ = 0.013, *p* = 0.911, Adj. *R* ^2^ = −0.056), and no interaction effect was found.

## 4 DISCUSSION

### 4.1 Summary of results

Here, we show that the invasive round goby (*N. melanostomus*) in the Port of Gothenburg likely originates from the western Baltic Sea and displays genotypic and phenotypic differences over short spatial scales across a steep salinity gradient. Fish caught in the Port of Gothenburg had the closest genetic relationship to conspecifics from the western Baltic Sea region, confirming our hypothesis that the spatially closest sites contribute the most with gene flow to the focus Port area (1). The relatedness was higher for fish sampled at the Outer port compared to fish sampled at the upstream sites. Genetic diversity was also higher in fish sampled at the high‐salinity (24.3 PSU) Outer port compared to the lower salinity (8.6 PSU) Inner port and Inner‐city port, supporting our hypothesis of genotypic differences between sites (2). When fish from sites differing in salinity were acclimated to freshwater (0 PSU) and seawater (30 PSU), SMR was not affected. However, fish sampled at the high‐salinity Outer port site showed a higher MMR irrespective of treatment. These fish also had fewer red blood cells and lower plasma calcium levels. Acclimation to either seawater or freshwater affected fish from both sites in the same way: seawater increased the blood osmolality and sodium levels, and freshwater increased the levels of the stress hormone cortisol. In summary, these results contrast to our hypothesis that fish from the Outer and Inner port would differ in their ability to acclimate to different salinities (3). However, they do support a scenario of sorting along the salinity gradient, based on either genotypic or phenotypic differences.

### 4.2 Genomic patterns of the introduction event and connectivity to other regions

Based on genomic analysis, round gobies from the Port of Gothenburg are significantly diverged from all other samples in this study. Fish from the Port of Gothenburg share the most similarities with conspecifics from the western Baltic Sea region. This is also the region that is closest geographically. Among sites from this region, fish from the harbour Port of Travemünde are most related to fish from Gothenburg. This fits with the general consensus that shipping transport is the most important vector for round goby spread (Kotta et al., 2016; la Rue et al., 2011). However, given its recent introduction to Gothenburg (first officially reported in 2010), Travemünde is too divergent to be the only source population for the Port of Gothenburg. In fact, Travemünde and the other samples in the western Baltic Sea are far more diverged from the Port of Gothenburg samples than from the geographically more distant and older central and northern Baltic Sea samples. When comparing between regions, the fish caught in the Port of Gothenburg show 19 private alleles, which supports that their source population may not have been included in this study. Ferry lines between the Port of Kiel (Germany) and Gothenburg run on a daily basis, and the species is known to be abundant in this port since 2006 (Neukamm, 2009). Other likely donor sites could be the industrial ports of Szczecin and Gdánsk (both in Poland), where the round goby was detected in the 1990s (Kornis et al., 2012; Sapota, 2004). In contrast, the most distantly related fish were from the rivers Elbe and Rhine in northern continental Europe. These populations have previously been described as belonging to a separate freshwater ‘ecotype’, ancestrally adapted to freshwater systems in the Ponto‐Caspian region (Green, Apostolou, et al., 2021). The genomic brackish ancestry of the Gothenburg fish likely limits them from rapid colonization of freshwater systems, which may explain the lack of fish caught further upstream the Göta Älv river (red sampling sites, Figure 1) and the higher levels of the stress hormone cortisol in fish when kept at 0 PSU. A previous study also found the highest level of heat shock protein (hsp70) expression when round gobies caught from Guldborgsund were kept in freshwater (compared to 10 and 30 PSU salinities) (Puntila‐Dodd et al., 2021). These mechanisms point to higher stress in freshwater for the round gobies with brackish ancestry, and a reason they will avoid it.

### 4.3 Genomic and phenotypic patterns within the salinity gradient explains sorting

Strong founder events are common among NIS (Prentis et al., 2008), also in marine environments (Flanagan et al., 2021; Hamner et al., 2007) where connectivity otherwise is higher due to life history strategies such as larval drift and lower landscape heterogeneity (Boström et al., 2011). The high genetic diversity (Tables S1 and S3) in fish from the Port of Gothenburg, only marginally lower than in other regions, suggests the population has experienced little‐to‐no founder effects, as previously seen in other introductions (e.g. when the species colonized the Great Lakes; Brown & Stepien, 2008). Genetic diversity was overall higher in the Outer port compared to the lower salinity Inner port sites. Individual clustering and *F*_*ST*_ estimates showed a stronger genetic connectivity between the Outer port and the other regions, compared to the inner sites and other regions. Although the Inner‐city port and the Inner port sites showed no differentiation, the Outer port was only significantly different from the geographically more distant Inner‐city port. Collectively, these results provide evidence for a stepping‐stone process, whereby round gobies most likely were introduced in the Outer port, a high‐intensity shipping area (Figure 1) sometime in the 2000s (SLU Swedish Species Information Centre, 2022), and have stepwise moved into the lower salinity environments upstream. The environmental heterogeneity is also likely to affect genomic signatures and could be a second source of the genomic divergence across the steep salinity gradient, and potentially help maintain it in the future (Figure 1). This sorting across an environmental gradient has previously been found in other NIS, most notably in terrestrial systems (Phillips & Perkins, 2019; Shine et al., 2011), but examples from marine environments also exist. For example, an invasive ascidian was recently found to be genotypically sorted based on depth, with salinity and temperature likely affecting their distribution patterns (Hudson et al., 2019).

Site‐specific differences are also evident in certain aspects of the species physiology. For example, blood Ca^2+^ content and haematocrit values were higher in fish caught from the low‐salinity Inner port site compared to the high‐salinity Outer port site, regardless of treatment in the common garden set‐up. The same was also true for MMR, which was consistently higher in the Outer port, regardless of salinity treatment. These subtle but evident physiological differences between sites may be attributed to long‐term ontogenetic acclimation (West‐Eberhard, 2003), genetic inheritance (Green, Apostolou, et al., 2021), or a combination of both (Czypionka et al., 2012). The adaptive benefits of higher MMR could potentially be explained by intraspecific ecology: more active and motile fish species have higher MMR compared to, for example, ambush predators (Norin & Clark, 2016). Higher Ca^2+^ is normally found in seawater acclimated euryhaline fish, but our data suggest this is a site‐specific difference, which was not influenced by the treatment. The mechanisms and potential adaptive value of these differences are unknown, but could be a reflection of unknown differences in prey abundance/species, community composition, and niche space and/or possibly be attributed to ecologically associated behavioural syndromes rather than osmoregulatory physiology. This again highlights the ecological differences between these spatially close sites.

No site‐specific responses to the salinity treatment in our common garden experiment were found for any of the measured traits. However, the salinity treatment did affect blood osmolality and Na^+^ values similarly for both sites. As in previous studies, this indicates that the adult physiology is affected by salinity differences and that potential acclimation to these conditions may be expected over the long term (Behrens et al., 2017). However, short‐term ontogeny, at least for adult round gobies, does not explain the site‐specific physiological differences observed. Rather, subtle genetic adaptation (Bernardi et al., 2016) or transgenerational plastic responses (Caño et al., 2016) may be involved. Phenotypic sorting, where the phenotypes carrying the more suitable genes are either selected to survive and reproduce, or congregate based on behavioural preferences, has been found in other NIS studied across environmental gradients (Bernardi et al., 2016; Hudson et al., 2019; Shine et al., 2011; Tepolt & Palumbi, 2015), and this could also be the case for round gobies in strong environmental gradients. Since round gobies can move several kilometres between brackish and fresh water (Christoffersen et al., 2019), behavioural sorting of round gobies based on phenotypic preference is possible in the ~10 km gradient of the Port of Gothenburg. The species also displays seasonal migration (Behrens et al., 2022), possibly selecting for the ability of homing in on certain environmental cues (Belanger et al., 2003). Furthermore, phenotypic differences attributed to local adaptation and environmental sorting based on phenotypic differences (albeit at much larger spatial scales) have previously been described for this species (Green et al., 2020; Green, Apostolou, et al., 2021).

### 4.4 Phenotypic patterns linked to risks of colonization of freshwater and fully marine environments

Since we did not see any site‐specific responses to salinity acclimation, we can expect that the adult round goby physiology, regardless of subtle genetic differences, is robust enough to accommodate these extremely different osmotic conditions. For example, in our common garden experiment, where fish were kept in either 0 PSU (freshwater) or 30 PSU (seawater), we detected no effect on SMR. Though differences might potentially be masked by our low samples sizes, our results differ from those by Behrens et al. (2017), which demonstrated an increase in the SMR of gobies at salinities deviating from isosmotic conditions. This is potentially explained by the ambient conditions the gobies are subjected to in their natural habitat, differences in salinity acclimation periods (i.e. 20 days in Behrens vs an average of 30 days in our study) and/or differences in water temperature (i.e. 18°C in Behrens vs 10°C in our study). Temperature increases metabolic rates for these ectothermic animals, and it is therefore likely that the physiological effects from salinity will become more pronounced in higher temperatures (Christensen et al., 2019; Morgenroth et al., 2019; Sardella et al., 2008), resulting in the observed differences between treatments. Despite differences in metabolic rates, Behrens et al. (2017) also found an increase in blood osmolality of gobies when held in seawater (30 PSU conditions) compared to freshwater (and indeed 0–25 PSU, a range where blood osmolality did not differ). Our measurements of gill NKA activity detected no differences across salinity treatments or sampling locations. Upregulating this enzyme or the associated ion channels is otherwise a common strategy for teleosts to deal with increases in salinity (Borgatti et al., 1992). However, for teleost families of marine ancestry where occurrence in freshwater is considered a derived trait (such as in Gobiidae), this may not be the case (Chubb et al., 1998). Similar to our results, the Hawaiian goby (*Stenogobius hawaiiensis*) showed no differences in NKA activity when subjected to a 10‐day exposure to 20 PSU and 30 PSU seawater compared to freshwater (McCormick et al., 2003). Together with the present study, this indicates that gobiidae may control osmoregulation using other mechanisms.

Similar to previous studies (Behrens et al., 2017), we show that adults of this species, despite the inability to fully accommodate changes in blood physiology, are tolerant to widely different salinity conditions. The moderately high levels of NKA activity throughout all the populations in the present study (ca. 6–7 μmol ADP mg^−1^ protein h^−1^) may allow for the apparent adaptive euryhalinity that the present study has shown. By maintaining high NKA activity throughout, the animal has a stronger potential to transfer into seawater at any time than it otherwise would, compared to, for example, anadromous salmonids with seasonal migration patterns tied to complex endocrinological feedback loops (McCormick, 1996). That said, the round goby is uncommon in the riverine environment upstream the Port of Gothenburg (Figure 1). This again highlights that the population‐genomic background and associated behavioural preferences for specific conditions are likely important for what environment this NIS spreads into.

The Port of Gothenburg is Scandinavia's largest cargo port, connected with direct cargo vessel lines to 65 trans‐national ports across the globe, on every continent, except South America (Port of Gothenburg, 2022). Unsurprisingly, all but 7 of these ports are located in temperate regions famous for marine invasions (Port of Gothenburg, 2022; Sardain et al., 2019). The risk of species introduction to and from this port is severe, and we advise management in other regions to allocate efforts to predicting the risk of round goby introduction, and locally to do the same for other high‐risk NIS. Environmental variables are already used to predict round goby spread (Bergkvist et al., 2020; Kotta et al., 2016), and eDNA monitoring has been proven to be a useful tool to establish the species presence and potentially biomass (Sundberg et al., 2021). With seascape genomics increasing in power and predictive capacity (Selkoe et al., 2016), NIS management can benefit greatly by adopting these approaches (Chown et al., 2015).

## 5 CONCLUSIONS

We show that the recently established round gobies in the Port of Gothenburg are genetically distinct from all other samples in this study. Least divergence was towards fish from the western Baltic Sea, which is likely one of multiple origins of this population. Within the salinity gradient, fish from the high‐salinity site and low‐salinity sites also differ in a number of phenotypic traits of potential adaptive value. These patterns are likely driven by multiple introductions into the Outer port site, and a stepping‐stone process of introduction as the species has continued to spread along the gradient. As the problem of NIS increases, detailed knowledge of seascape genomics and phenotypic characterization can benefit management even within an area as small as a coastal harbour inlet.

## CONFLICT OF INTEREST

The authors declare no conflict of interest.

## Supporting information


Supplementary Material

## Data Availability

Raw data supporting this study are available at Dryad digital repository https://doi.org/10.5061/dryad.gb5mkkws8. Benefits from this research accrue from the sharing of our data and results on public databases as described above (Green et al., 2022).
