# Supplementary material for: Invader at the edge — Genomic origins and physiological differences of round gobies across a steep urban salinity gradient
Source: Evol Appl. 2022 Jul 16;16(2):321–37. doi: 10.1111/eva.13437 (PMC9923490; doi:10.1111/eva.13437)

Supplementary information supporting

Invader at the edge - genomic origins and physiological differences of round gobies across a steep urban salinity gradient

Table S1. Genetic diversity of round goby (*Neogobius melanostomus*) from 12 937 SNPs. N= Sample size, Ar= Allelic richness, P(%) = Percent polymorphic sites, Pa = Private alleles, Ho = Observed heterozygosity, He = Unbiased expected heterozygosity, FIS = inbreeding coefficient.

Site	Region	N	Ar	P(%)	Pa	Hobs	Hexp	Fis
Outer port	Port of Gothenburg	26	1.49	63.7	0	0.18	0.19	0.036
Inner port	Port of Gothenburg	32	1.47	56.9	0	0.18	0.19	0.019
Inner-city port	Port of Gothenburg	30	1.46	55.8	0	0.18	0.19	0.008
Kindvig	Southern Baltic Sea	5	1.54	54.3	0	0.20	0.21	-0.06
Karrebaeksminde	Southern Baltic Sea	13	1.55	66.9	0	0.20	0.21	0.024
Guldborgsund	Southern Baltic Sea	32	1.57	73.0	1	0.21	0.22	0.024
Travemünde	Southern Baltic Sea	31	1.60	78.8	18	0.22	0.23	0.024
Mariehamn	Northern Baltic Sea	26	1.45	53.0	5	0.17	0.18	0.041
Turku	Northern Baltic Sea	27	1.56	72.4	44	0.20	0.21	0.033
Raahe	Northern Baltic Sea	22	1.52	67.1	1	0.19	0.20	0.046
Elbe	European rivers	29	1.55	72.4	491	0.21	0.21	0.021
Rhine	European rivers	32	1.46	69.2	856	0.17	0.17	0.021

Table S2. Lover left: Pairwise F_{ST} estimates of 12 samples of round goby (*Neogobius melanostomus*) using 12 937 SNPs, calculated according to Weir and Cockerham (1984). Bold values indicate significance of the estimates. Upper right: p-values as estimated using Fisher's exact tests with 5000 Monte Carlo replicates and false discovery rate corrections.

	Outer port	Inner port	Inner-city port	Kindvig	Karrebaeksminde	Guldborgsund	Travemünde	Mariehamn	Turku	Raahe	Elbe	Rhine
**Outer port**		0.708	0.008	0	0	0	0	0	0	0	0	0
**Inner port**	0.0208		1	0	0	0	0	0	0	0	0	0
**Inner-city port**	**0.0236**	0.0052		0	0	0	0	0	0	0	0	0
**Kindvig**	**0.1306**	**0.1661**	**0.1697**		1	1	1	0	1	0.886	0	0
**Karrebaeksminde**	**0.1195**	**0.1516**	**0.1586**	0.0013		1	0	0	0	0	0	0
**Guldborgsund**	**0.1243**	**0.139**	**0.1455**	0.0175	0.0108		0	0	0	0	0	0
**Travemünde**	**0.1091**	**0.1262**	**0.1295**	0.067	**0.0683**	**0.0616**		0	0	0	0	0
**Mariehamn**	**0.1851**	**0.2185**	**0.2185**	**0.1532**	**0.1466**	**0.1449**	**0.1432**		0	0	0	0
**Turku**	**0.1219**	**0.148**	**0.1534**	0.0724	**0.0752**	**0.0805**	**0.0687**	**0.1538**		0	0	0
**Raahe**	**0.1388**	**0.1657**	**0.1718**	0.0914	**0.093**	**0.0937**	**0.0818**	**0.1728**	**0.0973**		0	0
**Elbe**	**0.2054**	**0.2181**	**0.2237**	**0.169**	**0.1687**	**0.1598**	**0.1432**	**0.232**	**0.1601**	**0.1932**		0
**Rhine**	**0.3201**	**0.3319**	**0.3355**	**0.31**	**0.2965**	**0.278**	**0.2642**	**0.3421**	**0.2836**	**0.3055**	**0.233**	

Table S3. Results of AMOVA detailing degrees of freedom (Df), sum of squares (SS), mean of squares (MS) and estimated variance of each of the four regions, among the sites within those regions, among individuals within sites and within individuals.

Source	Df	SS	MS	%	p-value
Among Regions	3	157291	52430.322	9.8	0.001
Among Site Within Regions	8	103762.7	12970.339	8.9	0.001
Among Individuals Within Site	293	618522.1	2110.997	2.1	0.01
Within Individuals	305	612148	2007.043	79.2	0.001
Total	609	1491723.8	2449.464	100	

Table S4. Mean values of gill and blood samples from the invasive round goby (*Neogobius melanostomus*) caught from a high salinity site (outer port) or a low salinity site (inner port) and acclimated to either 0 PSU or 30 PSU salinity.

Tissue	Parameter	Site of catch	Salinity treatment	Mean value	CI-95%
Gill	Na+/K+-ATPase (NKA) activity	Outer port	30 PSU	6.20	4.68-7.72
		Outer port	0 PSU	7.18	4.80-9.56
		Inner port	30 PSU	7.33	5.76-8.89
		Inner port	0 PSU	6.80	4.14-9.46
Blood	Glucose	Outer port	30 PSU	3.24	1.92-4.56
		Outer port	0 PSU	3.45	2.84-4.06
		Inner port	30 PSU	2.83	2.12-3.54
		Inner port	0 PSU	3.36	2.54-4.17
Blood	Cortisol	Outer port	30 PSU	8.00	1.34-14.66
		Outer port	0 PSU	22.62	6.15-39.10
		Inner port	30 PSU	10.91	2.48-19.34
		Inner port	0 PSU	42.67	14.06-71.27
Blood	Potassium (K^+)	Outer port	30 PSU	1.24	0.51-1.97
		Outer port	0 PSU	1.32	1.07-1.57

		Inner port	30 PSU	1.46	1.17-1.75
		Inner port	0 PSU	1.26	1.05-1.47
Blood	Sodium (Na^+)	Outer port	30 PSU	171.30	164.33-178.27
		Outer port	0 PSU	169.85	165.42-174.29
		Inner port	30 PSU	179.88	168.07-191.69
		Inner port	0 PSU	166.47	161.35-171.59
Blood	Calcium (Ca^{2+})	Outer port	30 PSU	1.82	1.66-1.97
		Outer port	0 PSU	1.88	1.52-2.23
		Inner port	30 PSU	2.24	1.96-2.52
		Inner port	0 PSU	2.27	2.03-2.51
Blood	Haematocrit	Outer port	30 PSU	15.00	11.18-18.82
		Outer port	0 PSU	17.45	14.01-20.90
		Inner port	30 PSU	19.80	16.05-23.55
		Inner port	0 PSU	21.67	19.19-24.14
Blood	Osmolality	Outer port	30 PSU	346.75	336.75-356.75
		Outer port	0 PSU	341.00	333.53-348.47
		Inner port	30 PSU	353.60	344.16-363.04

Inner port	0 PSU	342.17	336.50-347.83

Figure S1. Map of sampling sites and regions of 305 round gobies (*Neogobius melanostomus*) compared in the study.

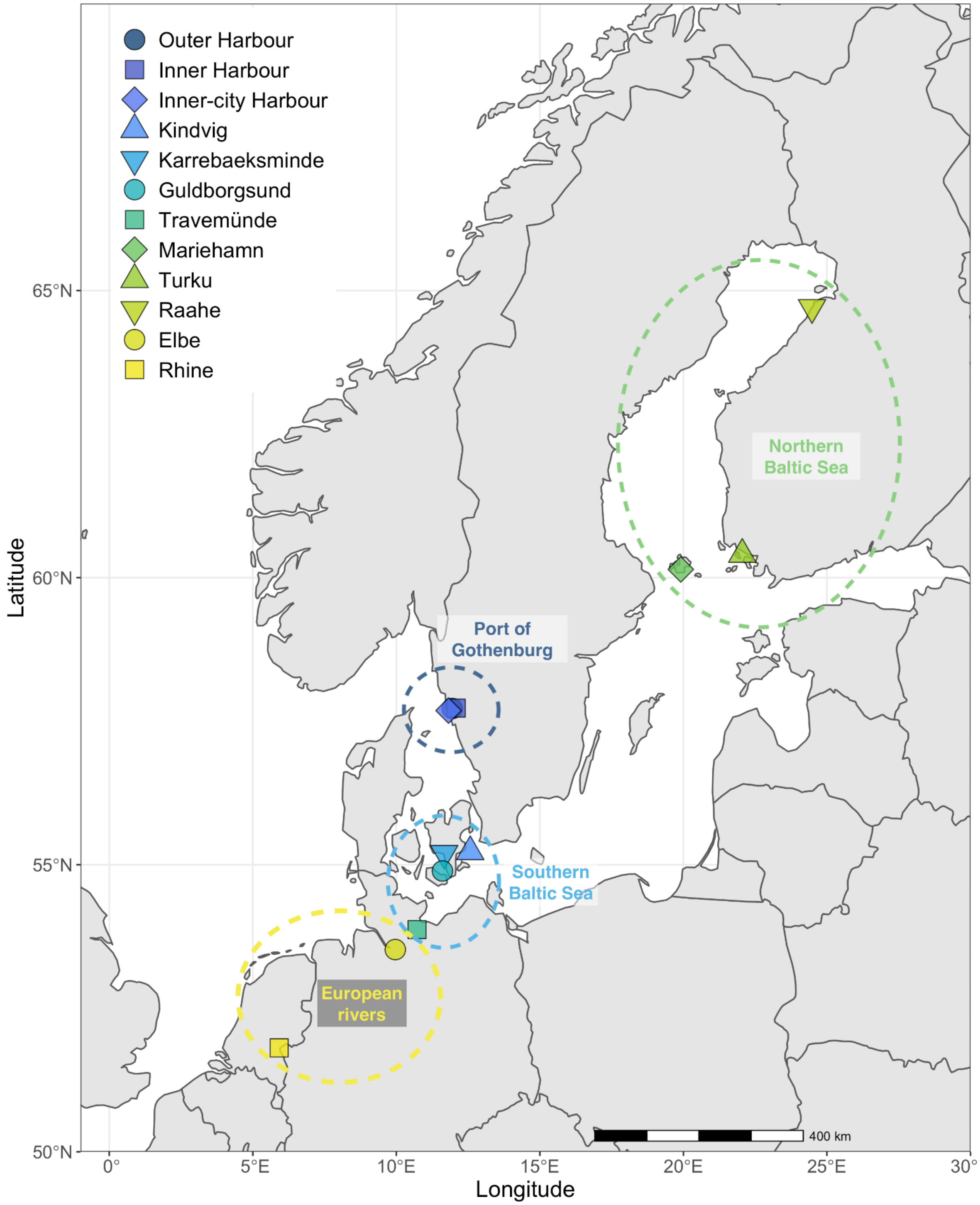

Figure S2. Illustration of the recirculation system set up during experimental treatments of the fish at the Zoology building at the Department of Biological and Environmental Sciences, University of Gothenburg. Yellow arrows indicate water flow and the green boxes foam filters. Eleven to seventeen round gobies (*Neogobius melanostomus*) were housed in each aquarium during experiments. Four of these systems were used, one for each treatment group (Outer port fish first kept in 15 PSU, then in 0 PSU; Outer port fish first kept in 15 PSU, then in 30 PSU; Inner port fish first kept in 15 PSU, then in 0 PSU; Inner port fish first kept in 15 PSU, then in 30 PSU).

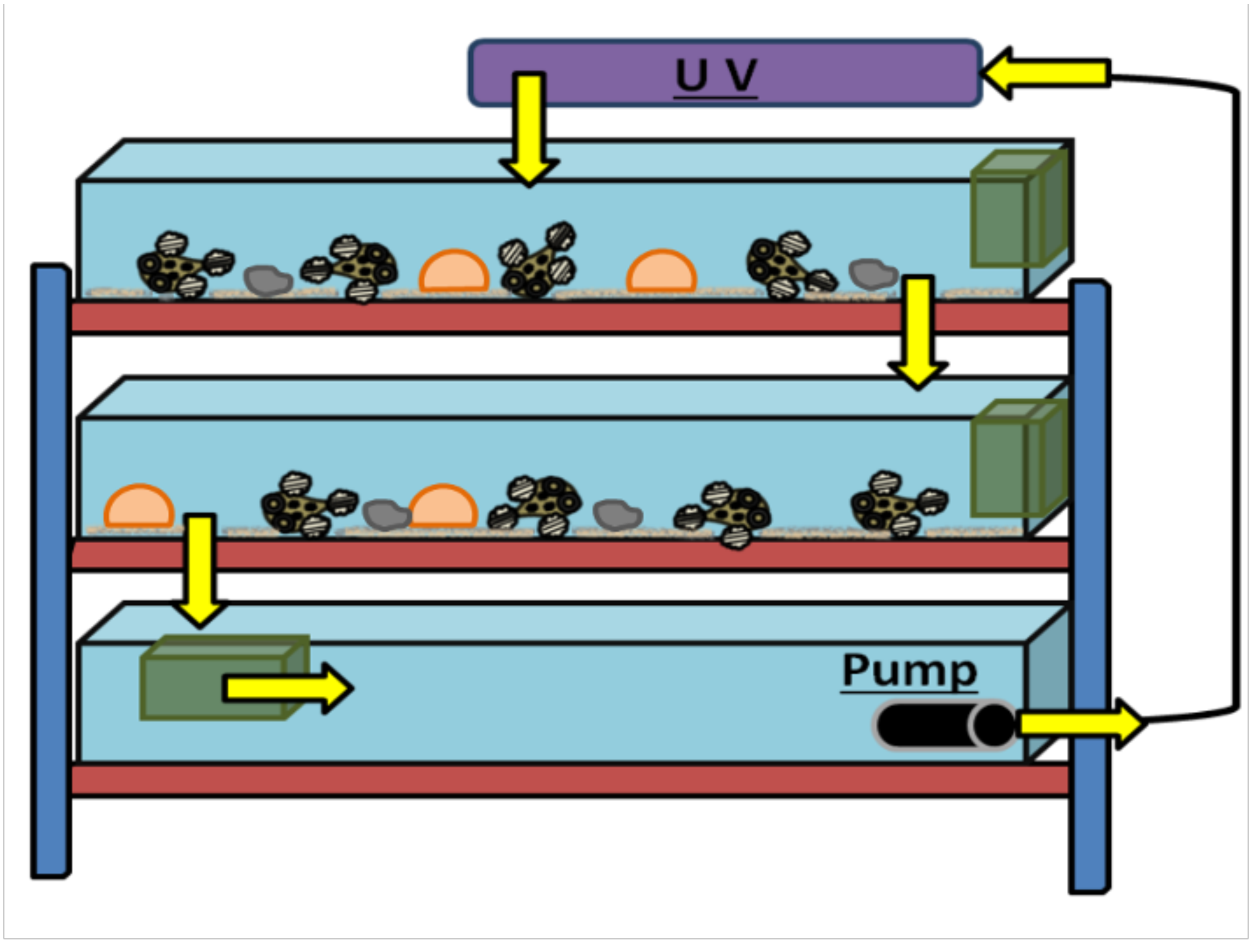

Figure S3. Values of the cross-entropy criterion as a function of the number of factors used in snmf() runs from the R package LEA. Calculated from 305 round gobies (*Neogobius melanostomus*) based on 12 937 SNPs.

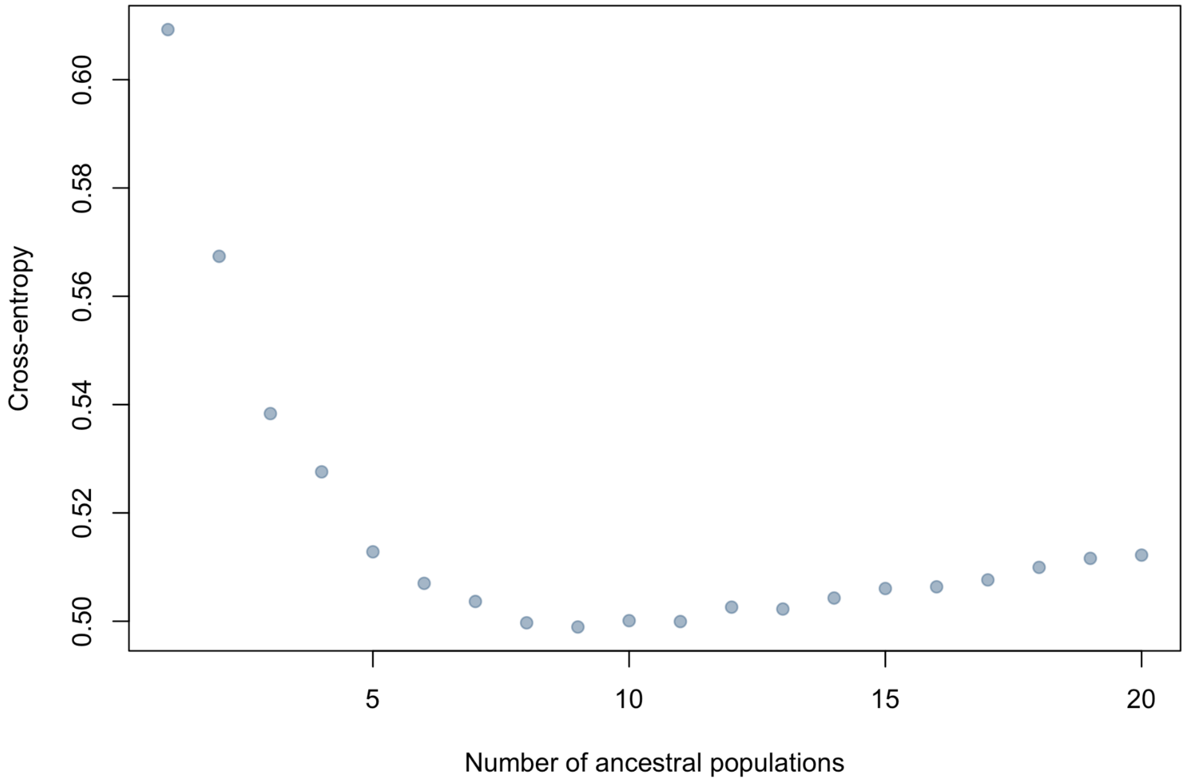

Figure S4. Individual ancestry of 305 round gobies (*Neogobius melanostomus*) based on 12 937 SNPs for K = 2-10 estimated using sNMF. Each vertical bar is one individual and the colour is the proportion of that individual assigned to the different K clusters. Individuals are separated by sampling sites and grouped in the four Baltic Sea regions.

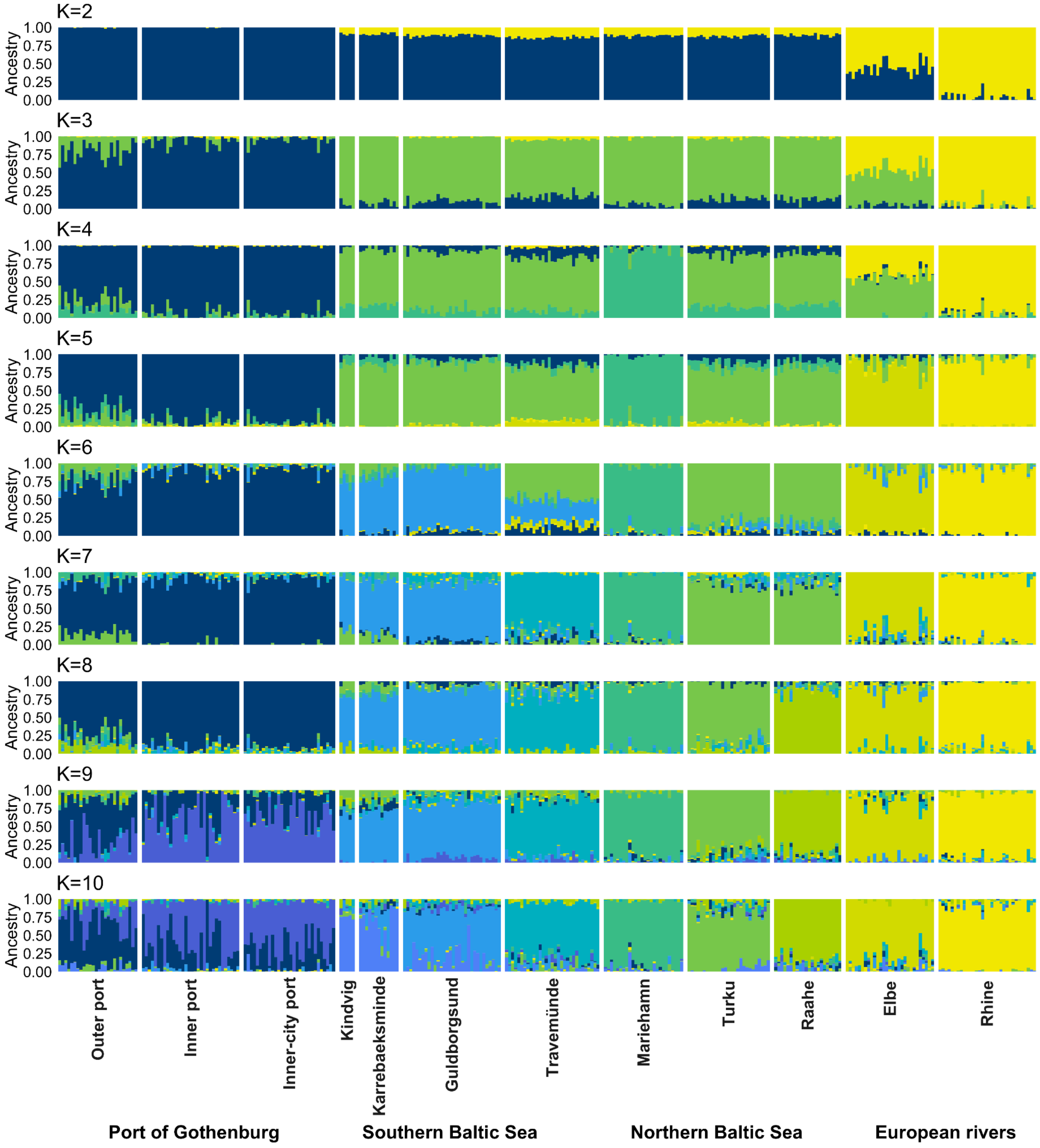

Figure S5. Visualisation of 3rd-8th principal component of a principal component analysis (PCA) on 305 round gobies (*Neogobius melanostomus*) genotyped at 12 937 SNPs. Each point represents one individual, colours represent sampling sites and shape is used for better distinction. Percentage of variation explained by each pc is displayed on each axis. Eigenvalues show the relative explanatory power of each component in ascending order.

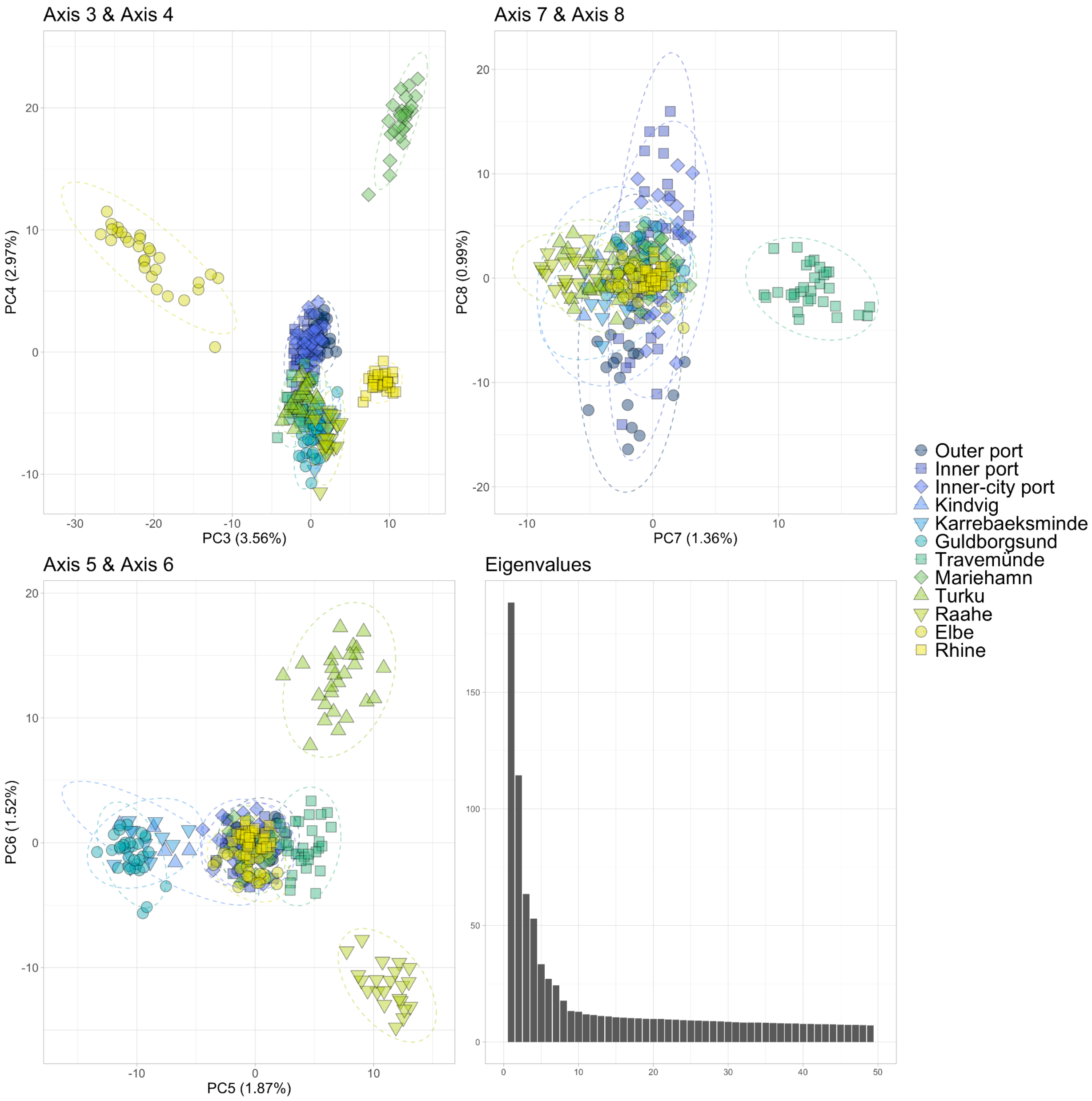

Figure S6. First (x-axis) and second (y-axis) component of a principal component analysis (PCA) on 88 round gobies (*Neogobius melanostomus*) from the Port of Gothenburg, genotyped at 12 937 SNPs. The first component explains 4.87% of the total variation and the second 3.04%. Each point represents one individual, colours represent sampling sites and shape is used for better distinction.

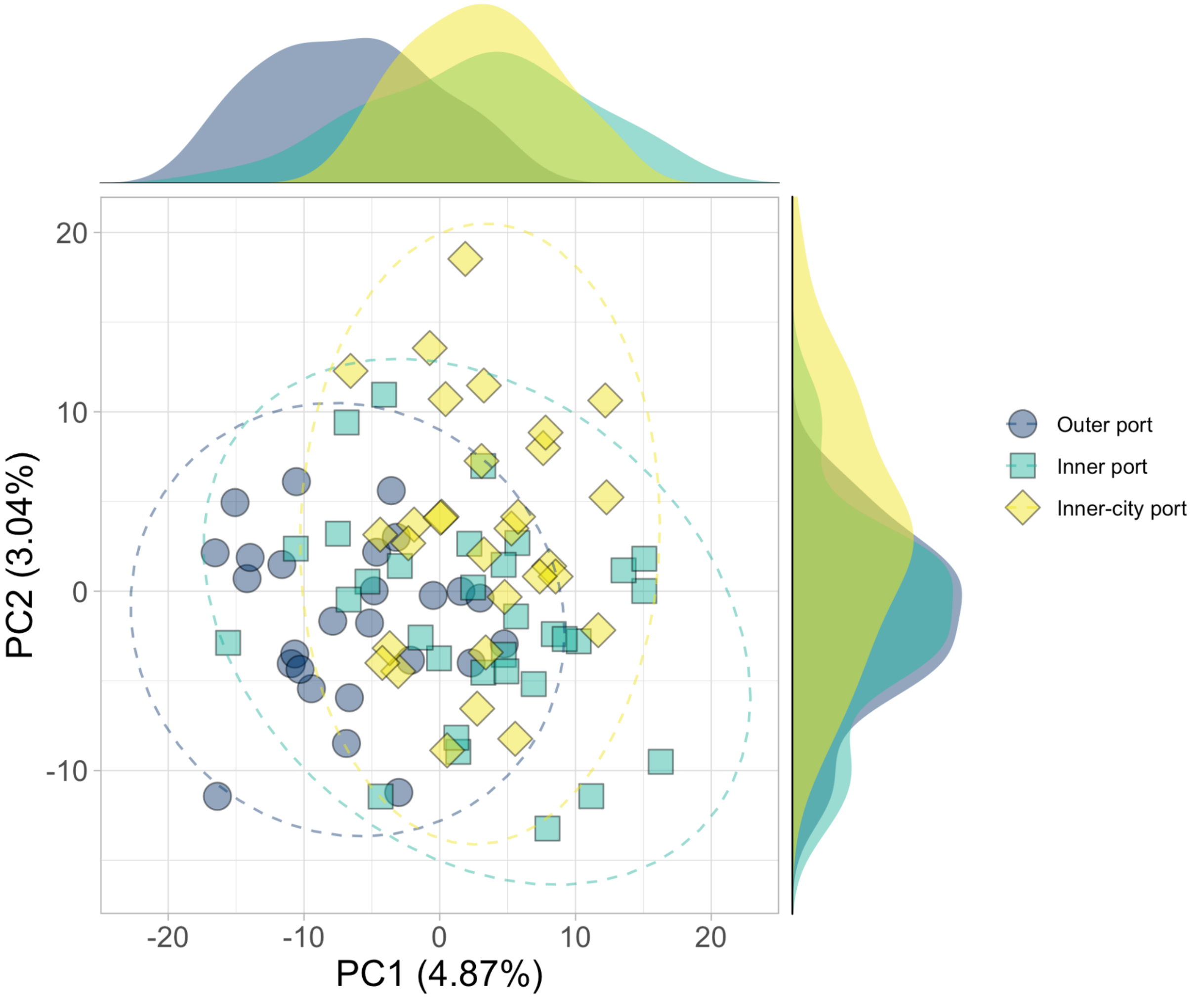

Figure S7. Principal component analysis PCA loading plots for neutral and outlier loci of 305 round gobies (*Neogobius melanostomus*) genotyped at 12 937 SNPs.

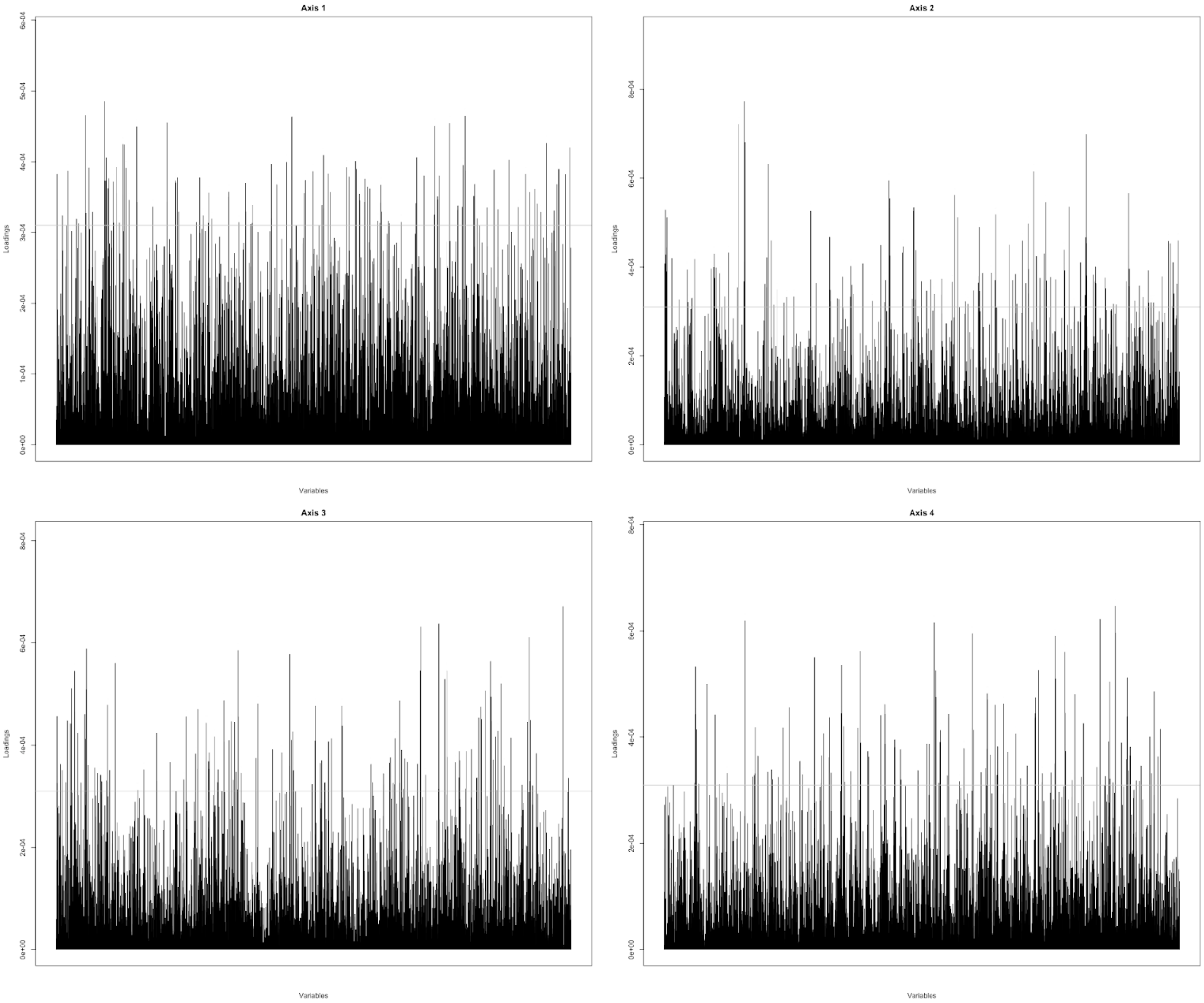

Figure S8. Visualisation of 1st-8th principal component of a principal component analysis (PCA) on 305 round gobies (*Neogobius melanostomus*) genotyped at 12 540 neutral SNPs (the dataset after 395 outlier SNPs were identified using Pcadapt, and then removed). Each point represents one individual, colours represent sampling sites and shape is used for better distinction. Percentage of variation explained by each pc is displayed on each axis. Eigenvalues show the relative explanatory power of each component in ascending order.

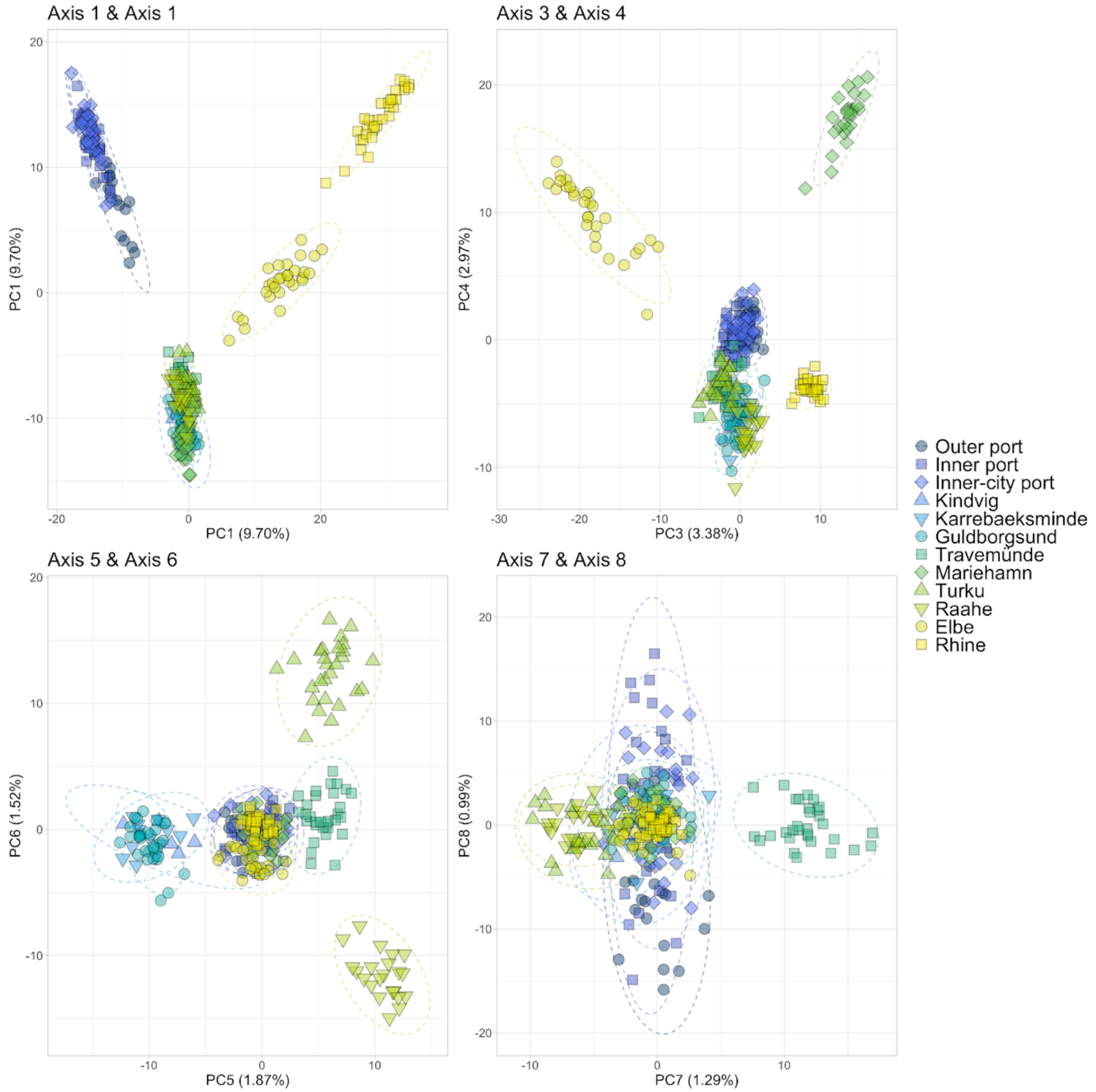

Supplement: Supplementary file 1 — Supplementary Material [file EVA-16-321-s001.pdf]
